# Recent Developments in Inertial and Centrifugal Microfluidic Systems along with the Involved Forces for Cancer Cell Separation: A Review

**DOI:** 10.3390/s23115300

**Published:** 2023-06-02

**Authors:** Alireza Farahinia, Wenjun Zhang, Ildiko Badea

**Affiliations:** 1 Department of Mechanical Engineering, University of Saskatchewan, Saskatoon, SK S7N 5A9, Canada; 2 College of Pharmacy and Nutrition, University of Saskatchewan, Saskatoon, SK S7N 5E5, Canada

**Keywords:** circulating tumor cells (CTCs), cancer metastasis, cell enrichment, cancer diagnosis, microfluidic-based cell separation approaches, lab-on-a-chip (LOC), lab-on-a-CD (LOCD)

## Abstract

The treatment of cancers is a significant challenge in the healthcare context today. Spreading circulating tumor cells (CTCs) throughout the body will eventually lead to cancer metastasis and produce new tumors near the healthy tissues. Therefore, separating these invading cells and extracting cues from them is extremely important for determining the rate of cancer progression inside the body and for the development of individualized treatments, especially at the beginning of the metastasis process. The continuous and fast separation of CTCs has recently been achieved using numerous separation techniques, some of which involve multiple high-level operational protocols. Although a simple blood test can detect the presence of CTCs in the blood circulation system, the detection is still restricted due to the scarcity and heterogeneity of CTCs. The development of more reliable and effective techniques is thus highly desired. The technology of microfluidic devices is promising among many other bio-chemical and bio-physical technologies. This paper reviews recent developments in the two types of microfluidic devices, which are based on the size and/or density of cells, for separating cancer cells. The goal of this review is to identify knowledge or technology gaps and to suggest future works.

## 1. Introduction

Cancer research and treatment have made significant progress in recent years, thanks to advancements in technology and scientific understanding. Listed below are some updates on the current state of cancer research and treatment:

- Immunotherapy: Immunotherapy is a type of cancer treatment that stimulates the body’s immune system to fight cancer cells. There has been a significant advancement in the use of immunotherapy, including the approval of several immunotherapy drugs for the treatment of various types of cancers [1,2], and a combined treatment with ablation [3,4].
- Precision medicine: Precision medicine is a personalized approach to cancer treatment, which involves the use of genetic and other molecular profiling to identify the unique characteristics of a person’s cancer [5,6]. This approach has led to the development of targeted therapies that are tailored to an individual’s cancer [7,8,9,10].
- Gene editing: Gene editing is a technique that allows scientists to modify the DNA of cells. This technology has been used in cancer research to develop new treatments and to improve the effectiveness of existing treatments [11,12].
- Liquid biopsies: Liquid biopsies are non-invasive tests that can detect cancer by analyzing blood or other body fluids. This technology has the potential to revolutionize cancer diagnosis and treatment, as it allows doctors to monitor cancer progression and response to treatment in real-time [13,14,15,16].
- Artificial intelligence: Artificial intelligence (AI) is being used in cancer research to analyze large amounts of data and to develop more effective cancer treatments. AI can also help doctors make more accurate diagnoses and develop personalized treatment plans for cancer patients [8,17,18,19,20].

Despite these advancements, cancer remains a major public health challenge, with new cases and deaths from cancer occurring every year. As the second-highest cause of death in the world after heart disease, cancer accounts for one in four deaths [21]. The American Cancer Society (ACS) predicted that 10 million of 16 million cancer-infected people would surrender to this disease (roughly 28,000 deaths each day) [22]. Research continues to identify new targets for cancer treatment and to develop more effective therapies. Additionally, efforts to improve cancer prevention, early detection, and access to quality care are ongoing.

Cancer forms its deadliest shape when circulating tumor cells (CTCs) [23,24] separate from the original tumor or cancer tissue and spread throughout the body in the bloodstream or lymph alongside healthy hematological cells [25,26]. From this stage onward, the patient would encounter drastic effects [27]. Consequently, numerous attempts have been made to control the cancer development because timely diagnosis plays a vital role in ensuring various treatment options are available. Therefore, the first step to controlling cancer development and providing the best treatment is to know the characteristics of CTCs, as they are different from one person to another and from one type of cancer to another. To investigate CTC genetics, CTCs must be first detached from the other blood cells. However, the salient feature is that the concentration of CTCs in blood samples is extremely low (1–10 CTCs per mL of whole blood in patients with metastatic disease [28]), which leads to the problem of an insufficient number of CTCs for further downstream analysis. Numerous methods have been developed to improve CTC enrichment, detection, sensitivity, and purity. These methods are mostly based on the physical properties and diverse molecular biomarker profiles of CTCs. Indeed, these methods are introduced based on the different biochemical and physical properties of CTCs compared to the other blood cells [29]. Most CTCs have different surface proteins compared to blood cells, and their average size is larger than the largest white blood cells (WBCs) and red blood cells (RBCs) [30].

As mentioned earlier, one of the current approaches to cancer treatment is precision medicine, which aims to tailor treatment to individual patients based on their specific genetic and other molecular characteristics [8,10]. This approach requires the ability to analyze the genetic and molecular makeup of tumors and monitor the response to treatment; however, this investigation can only be performed when a sufficient amount of cancer cells, which have been rapidly and accurately separated from other blood cells, is available. This is where microfluidic devices come in. Microfluidic devices are small, lab-on-a-chip devices that provide a controlled environment for the separation of CTCs and facilitate the prediction of their behaviors, reduce the sample quantity, and minimize the diagnostic time and treatment cost [31,32,33]. It is worth mentioning that traditional clinical procedures can be scaled down to the microscale by mimicking macroscale processes and applying various external forces [34,35]. A microfluidic system, as an example, allows researchers to explore the behaviors and responses of micro/nanoparticles toward these applied forces. Due to their small size and miniaturization, microfluidic devices allow us to efficiently manage and observe testing. The main advantage of such devices is dealing with accurate fluid flow management in microliters (10^−6^ L) to picolitres (10^−12^ L) within microvolume channels [36], which elevates their medical usage [37,38,39]. The size reduction in the conventional drug delivery devices to micro- and nano-scales makes it possible to insert them into patients’ bodies. As such, their health situation can be checked meticulously at any moment. Moreover, doctors can devise preventive remedies outside the patients’ bodies and apply them inside. These devices can inject drugs at different doses continuously or intermittently over prescribed time intervals. In conventional drug delivery types, the medicine may enter the body at higher or lower doses than required or at an inappropriate rate, resulting in toxicity or low efficiency. They are usually made from polymers such as polyethylene glycol diacrylate (PEGDA) [40], parylene [41], and polydimethylsiloxane (PDMS) [42,43], which are widely used in biomedical applications such as cell separation and mixing, 3D bioprinting [44], and organs-on-a-chip (OOC) [45,46].

Other examples of their applications are (1) extensive use in capillary electrophoresis, isoelectric centralization, immunoassay, flow cytometry, sample injection in mass spectrometry, PCR amplification, DNA analysis, cell isolation and manipulation, and cellular modeling; (2) chiefly related to studying bacteria that are resistant to antibiotic drugs, the transfer of nanoparticles in the blood, and the kinetic investigation of chemical reactions; (3) diagnostic usage, including cancer and pathogen detection; (4) measuring the molecular diffusion coefficient, viscosity, fluid alkalinity, and the coefficients of chemical bonding; (5) in biological products to improve and control medicinal proteins and experiments containing human cells [47]; (6) in the medical industry to separate, classify, and sort cells [48] due to the accurate measurement of stem cells, overall efficiency enhancement, better fluid management, and precise biological simulation [49].

During cancer diagnosis and treatment, microfluidic devices are used for a variety of purposes, such as:

- Liquid biopsy: Microfluidic devices can isolate and analyze circulating tumor cells and cell-free DNA from a patient’s blood, allowing for non-invasive cancer diagnosis and monitoring [50,51].
- Drug screening: Microfluidic devices can be used to screen large numbers of drugs for their effectiveness against specific cancer types, allowing for more efficient drug discovery [52,53,54,55].
- Tumor microenvironment analysis: Microfluidic devices can be used to recreate the tumor microenvironment in vitro, allowing researchers to study how tumors interact with their surroundings and the development of new cancer therapies [56,57,58].
- Personalized medicine: Microfluidic devices can be used to test the effectiveness of different cancer treatments on a patient’s cancer cells, allowing for the development of personalized treatment plans [59,60].

Microfluidic technologies can address some of the limitations in the current cell separation methods, such as fluorescence-activated cell sorting (FACS) or magnetic-activated cell sorting (MACS) [61]. For example, (1) current cell separation methods are often limited in terms of the number of cells that can be processed at once, which can be a bottleneck in many applications. Microfluidic devices can overcome this limitation using high-throughput microchannels and parallelization to sort large numbers of cells quickly and efficiently [62]. (2) Current cell separation methods can result in low purity due to non-specific binding or incomplete separation. Microfluidic devices can use a combination of physical and chemical methods to achieve higher purity, such as using specific antibodies or binding surfaces to selectively capture and release target cells [63,64,65]. (3) Current cell separation methods can be expensive due to the need for specialized equipment and reagents. Microfluidic devices can be designed to be cost-effective using low-cost materials and simple fabrication techniques, such as 3D printing, molding, laminating, and high-resolution nanofabrication [66,67,68]. (4) Traditional cell separation methods can be invasive or damaging to cells, which can affect their viability and function. Microfluidic devices can use gentle and non-invasive methods to sort cells, such as hydrodynamic forces, that minimize damage to cells and preserve their integrity [69]. (5) Current methods for cell separation may not be able to sort all cell types, such as rare or fragile cells. Microfluidic devices can be designed to handle a wide range of cell types and sizes, including circulating tumor cells, stem cells, and immune cells. Recent microfluidic technologies use different physical mechanisms, including filtration [70], hydrodynamic [71], inertial [72], deterministic lateral displacement [73,74], pinched flow fractionation [75], and centrifugation [76], to facilitate the separation process. These methods, which are categorized into passive separation techniques, can separate target cells from a heterogeneous cell population by exploiting differences in the properties of cells, including their size, density, shape, deformability, and compressibility properties [77,78,79,80,81,82].

Overall, microfluidic devices offer a promising approach to overcoming many of the limitations in the current cell separation methods, resulting in higher purity, throughput, and viability of sorted cells. They also have the ability to advance cancer research and treatment with the potential to improve diagnosis, drug discovery, and personalized medicine. However, further research is needed to optimize these devices and fully realize their drawbacks, limitations, and potential in cancer diagnosis and separating cancer cells. Indeed, although microfluidic devices have many potential benefits for cancer research, their cost and complexity can limit their accessibility for certain populations. Researchers and manufacturers need to be aware of these limitations and work to address them in order to maximize the potential of microfluidics for a wide range of applications. Some of their main limitations are (1) microfluidic devices can be expensive to develop and manufacture, which can limit their accessibility for certain populations [83]. For example, low-income communities or developing countries may not have the resources to invest in expensive equipment or technologies. (2) Microfluidic devices are often complex and require specialized knowledge to design and operate [84,85]. This can be a barrier for users who are not familiar with microfluidics or do not have access to trained professionals. (3) Microfluidic devices can be sensitive to environmental factors such as temperature, humidity, dust, and contamination [86,87]. This can lead to variability in results or even failure of the device, which can be frustrating for users. (4) Microfluidic devices are typically designed to handle small sample sizes, which may not be sufficient for some applications. This can limit the usefulness of microfluidics in certain fields, such as clinical diagnostics or drug development. (5) Microfluidic devices can be difficult to reproduce consistently, which can be a challenge for researchers and manufacturers. Small variations in device design, fabrication, or operation can result in significant differences in performance, which can make it difficult to compare results between different studies or even between different devices.

This paper aims to present a detailed analysis of two common passive techniques for CTC separation in micro-scale dimensions. After the Introduction section, the rest of this paper is structured in the following way. Section 2 introduces the inertial isolation technique with a focus on different geometries and their advantages and limitations. Section 3 presents the centrifugal isolation technique and discusses the combination of this technique with other technologies. These combinations are a proper attempt to address the limitations in microfluidic systems for controlling fluid flow and avoiding channel clogging. Section 4 introduces the forces that are involved in particle/cell movement in a microfluidic system. In the end, Section 5 provides the conclusions of this review.

**Scheme 1:**
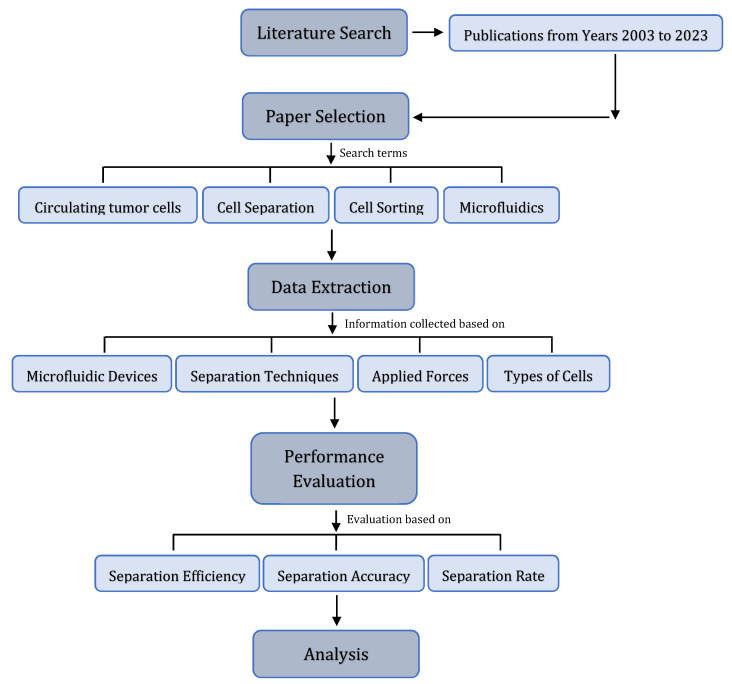
Overview of the research process and data collection methods used in this paper.

The flowchart in Scheme 1 provides a simple overview of the study process used in this paper. Of course, the actual process was more complex and involved additional steps, but this should give readers a general idea of how the authors conducted this review.

During the literature search, the authors conducted a comprehensive search of various scientific databases to identify relevant papers on microfluidic approaches to CTC separation and blood cell sorting. After screening the papers based on their titles and abstracts, those that were relevant to the topic were selected. The authors then generated and used a data extraction form to collect information on the microfluidic devices, separation techniques, types of applied forces for the separation, and types of cells that were separated or sorted in each study. Then, for the performance evaluation, they evaluated the performance of each microfluidic device based on metrics such as throughput, separation efficiency, separation accuracy, and separation rate. Finally, the authors analyzed and synthesized the collected data to provide an overview of the various microfluidic approaches that have been developed for CTC separation and blood cell sorting.

## 2. Inertial Microfluidics

### 2.1. Introduction

The conventional process for cell/particle separation was significantly updated after the invention of inertial microfluidics [88]. It has evolved into the predominant trend in sample preprocessing due to its high throughput, low cost, and straightforward control. Inertial microfluidics is an outstanding candidate for isolating rare CTCs from a blood sample or other target cells because it uses the hydrodynamic inertial effects of microfluidics and particle manipulation functions (focusing, separation, and capturing) with minimal sample volume at a low cost.

### 2.2. Particle Focusing in Straight vs. Curved Microchannels

**Figure 1:**
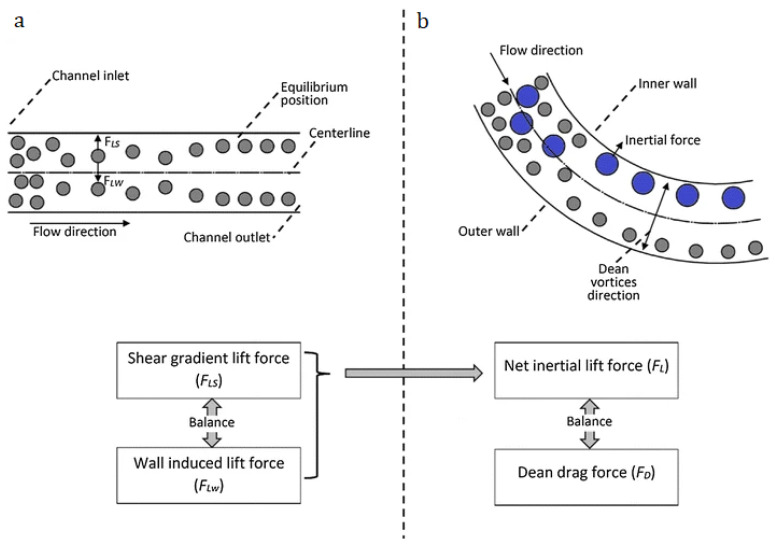
Particle/cell migration in a straight and curved channel: (**a**) In a straight channel, $F_{LS}$ pushes particles toward the channel walls, and $F_{LW}$ repulses them back toward the channel center line; (**b**) In the spiral channel, the balance between $F_{L}$ and $F_{D}$ causes particle migration to the equilibrium position [91] (Reprinted/adapted with permission from Ref. [91]. 2017, Springer Nature).

Inertial microfluidics was initially invented in a straight microchannel to sort and focus particles at their equilibrium positions (between the centerline and walls). The particle focusing mechanism works based on the hydrodynamic inertial forces on particles flowing inside a microchannel. It pulls the particles away from the walls and places them in an equilibrium position at specific cross-sectional positions in the channel. Such behavior is governed by channel geometry and flow conditions under the inertial forces in the microchannel [89]. A balance of two dominant forces in a straight channel will draw particles from the walls to the equilibrium position [90]. These two forces, i.e., the shear gradient inertial lift force $\left(F_{LS}\right)$ and wall-induced inertial lift force $\left(F_{\mathrm{LW}}\right),$ which are composed of the inertial lift force $(F_{L}=f_{L}\rho U^{2}d^{2}/D_{h}^{2}$, where $f_{L}$ is the dimensionless lift coefficient and d is particle diameter), will be thoroughly discussed in Section 4. However, as a brief introduction, $F_{LS}\;$ and $F_{\mathrm{LW}}$ are induced by the velocity gradient of the Poiseuille flow and the particle’s interaction with the nearby wall, respectively. $F_{LS}\;$ pushes the particles away from the microchannel centerline toward the walls, whereas $F_{\mathrm{LW}}$ drives them away from the walls toward the center. Finally, particles with varied sizes settle at different equilibrium positions between the centerline and walls where these two opposite inertial lift forces on the particle are balanced (see Figure 1a) [91]. The geometry of the microchannel cross-section affects the equilibrium positions of particles. For example, particles are gathered into an annulus with a radius of 0.6r (r is the channel radius) in a cylindrical channel [92]; or migrate to the four equilibrium positions near the centers of the walls in a square channel [93]; or first, move toward the two long walls and then become gently focused to the two equilibrium positions near the middle points on the long walls in a rectangular channel (the aspect ratio of the channel < 1) [94].

Figure 1b shows that adding curvatures to the flow channel can generate secondary vortices (i.e., Dean vortices) perpendicular to the main flow stream [95]. Indeed, curved channels cause velocity mismatches between liquid elements closer to the channel walls and those near the channel center [72,96]. Liquid elements close to the channel center have higher inertia, while the elements near the channel walls are relatively inactive. This velocity mismatch leads to two symmetrical secondary flows perpendicular to the liquid main flow. As a result, particles in spiral-shaped channel inertial microfluidics will follow these vortices in addition to the main flow. As shown in Figure 1b, by adjusting the dimension and shape of the channel, large particles can be concentrated near the channel’s inner wall while smaller particles flow near the channel’s outer wall [97,98].

Although microfluidic systems are typically performed using the Stokes regime with negligible fluid inertia and Reynolds number, inertial microfluidics works in an approximate Re range from 1 up to 100 (between the Stokes regime and turbulent regime) [96]. Therefore, particles migrate with fluid flow in such microfluidic channels due to the effect of the fluid’s inertia and viscosity. In this regime, these two effects on suspended particles, i.e., inertial migration and secondary flow, are related to finite inertial forces (see Figure 1). Indeed, Dean vortices are developed when randomly dispersed particles with different sizes are injected and flow through a spiral. The resultant drag forces make the particles follow the direction of these vortices in addition to the mainstream flow. It was proved that the strength of such vortices depends on the Dean number (De), Reynolds number (Re), and channel aspect ratio (AR) [96].

### 2.3. Inertial Microfluidic Devices with Different Geometric Designs

**Figure 2:**
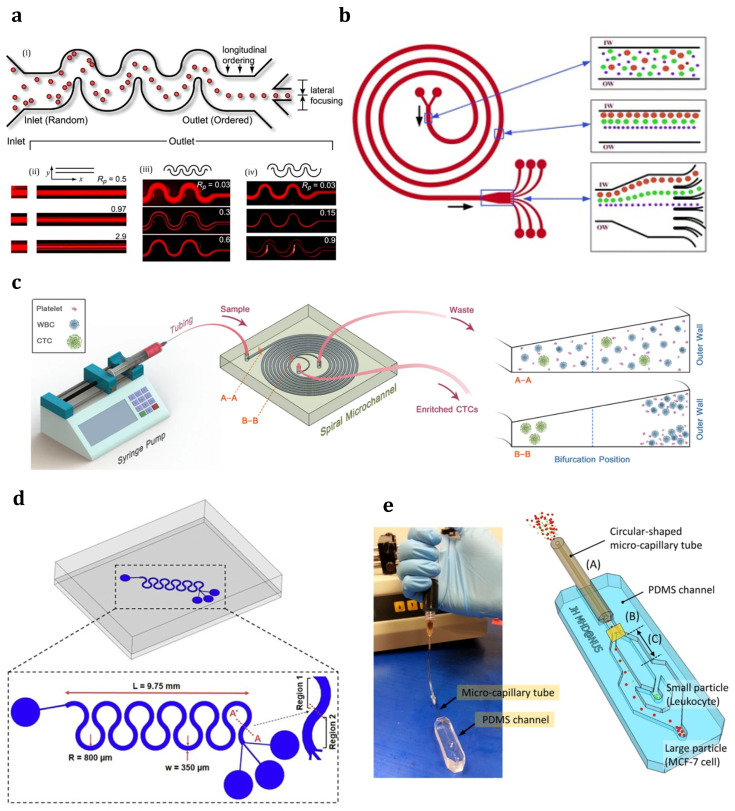
Inertial microfluidic separators in (**a**) a serpentine structure [99] (Reprinted/adapted with permission from Ref. [99]. 2007, National Academy of Sciences, U.S.A.); (**b**) a spiral structure [102] (Reprinted/adapted with permission from Ref. [102]. 2009, Royal Society of Chemistry); (**c**) a trapezoidal spiral channel microfluidic device [106]; (**d**) a symmetrically curved channel microfluidic device [107]; (**e**) a hybrid capillary-inserted microfluidic device [(A–C) indicates the first stage for viscoelastic 3D focusing at the inlet of the micro-capillary tube, the first bifurcation in the channel for initial separation of all particles, and the second stage of microchannel designed for viscoelastic separation, respectively.] [108] (Reprinted/adapted with permission from Ref. [108]. 2015, AIP Publishing).

In comparison to other microfluidic devices, inertial microfluidic systems perform at considerably higher Reynolds numbers. As opposed to external forces, using inertial effects such as the inertial lift force and Dean flow in inertial microfluidics results in high throughput and continuous cell sorting. Movement differences due to cell size and deformability have been used in inertial microfluidic devices with a variety of geometries such as straight, spiral, and multi-orifice structures to separate CTCs. Figure 2a displays the device with a serpentine geometric design, which was introduced by the Di Carlo Group in 2007. After the particles with an irregular arrangement are entered into the fluid, they will be finally ordered along the flow direction [99]. The Di Carlo group then investigated the effect of the fluid Reynolds number and particle size and density on the separation of deformable particles. They also tried to introduce a design with multi-stage separation to achieve a higher purity [100]. Recently, Mahboubidoust et al. presented the development of a hybrid acousto-inertial microfluidic platform with a serpentine microchannel with four different configurations for the separation of CTCs from neutrophils in whole blood [101]. The platform combines acoustic radiation forces and inertial microfluidics to achieve high efficiency and specificity for cell separation. The device consists of a piezoelectric transducer and a microfluidic channel with a series of curved pillars that induce Dean flow and particle migration. The acoustic radiation forces generated with the transducer are used to selectively trap and separate CTCs from neutrophils based on differences in their physical properties such as size and density. The performance of the device was evaluated and showed a high separation efficiency of 99.3% for MCF-7 cells and a high purity of 93.5% using standing surface acoustic waves (SSAWs).

Later, spiral geometry was chosen as a popular structure for microfluidic devices after trying to make a variety of geometries (Figure 2b) [102,103]. This type of structure benefits from simplicity, high efficiency, shorter length, and better controllability [104]. In addition to the low possibility of clogging and blocking in such channels, their separation efficiency and flow rate are high, which originates from the relatively large dimensions and lack of obstacles. Moreover, to improve the final accuracy, antibody indicators as a common diagnostic test can be embedded in this design [102]. Recently, Warkiani et al. described a label-free spiral microfluidic chip for the size-based separation of CTCs from a 7.5 mL sample under hydrodynamic forces in less than 40 min with an isolation efficiency of 85%. It was also shown that stacking three chips together delivered better results by separating CTCs from 7.5 mL samples in under 10 min [98]. Later, Thanormsridetchai et al. reported a 90% capture efficiency after developing a microfluidic device with five spiral microchannels to isolate CTCs [105].

In spiral channels, flowing particles within a curved subsection will experience lift and drag forces (caused by Dean vortices), which results in particle centrifugation [96]. In addition, the Dean vortices rotate in the same direction inside each subsection because the direction of the spiral–channel curvature is either outward (toward the platform edge) or inward (toward the platform center). As particles relocate from one point to another in the spiral, the Dean drag force changes due to changing the spiral curvature. However, it should be mentioned that this change is negligible due to relatively small spirals. Therefore, the inertial force keeps particles in a specific cross-sectional equilibrium position. This equilibrium position corresponds to the particle size under the effect of the Dean drag force. In other words, the ratio of inertial force to the Dean drag force specifies the relative position of differently sized particles to be focused inside the channel. As this ratio approaches zero, the Dean drag force is dominant. This condition is valid for particles with a size much smaller than the channel hydraulic diameter. Under such a circumstance, the Dean force drives small particles near the channel’s outer wall (close to the platform edge). In contrast, as the ratio approaches ∞, the dominant force for large particles with a diameter similar to the hydraulic diameter of the channel is inertial force, which makes large particles flow close to the curved channel’s inner wall (close to the platform center).

**Figure 3:**
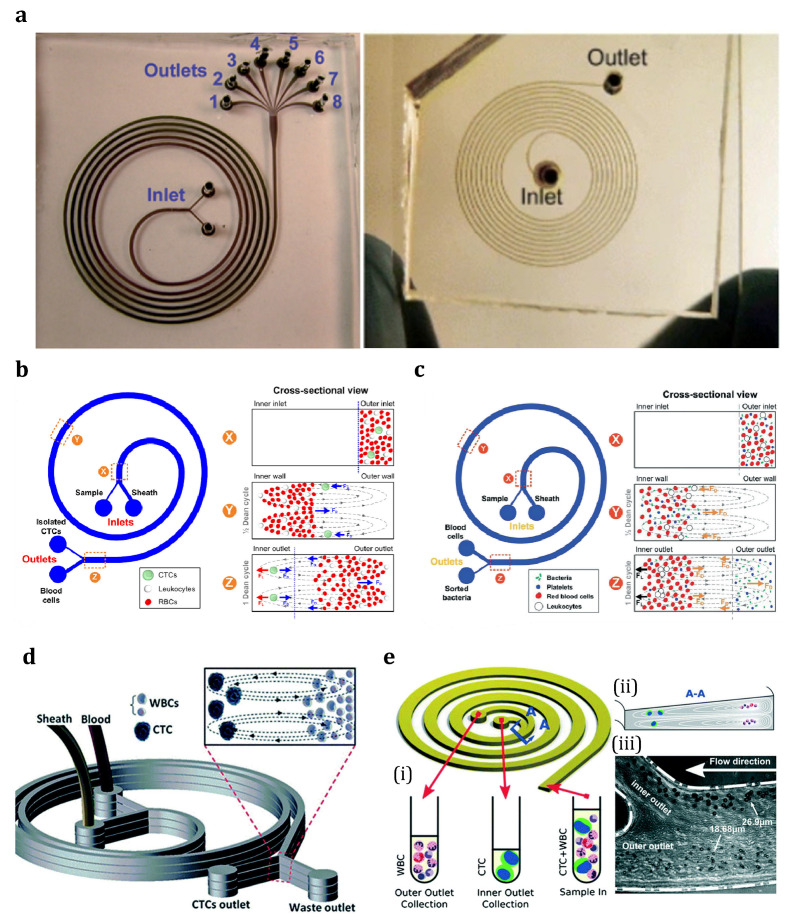

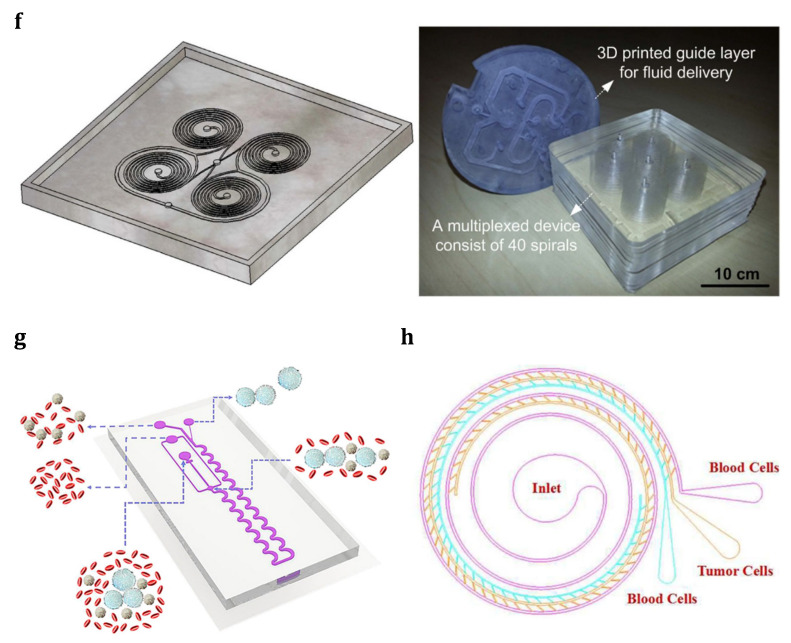
A spiral structure as a passive cell separation technique: (**a**) Five-loop and ten-loop spiral microchannels [95,102] (Reprinted/adapted with permission from Ref. [95]. 2008, Royal Society of Chemistry); (**b**) Schematic illustration showing DFF for CTCs isolation [112]; (**c**) Schematic illustration showing DFF for pathogen separation from blood [109] (Reprinted/adapted with permission from Ref. [109]. 2015, Royal Society of Chemistry); (**d**) A multi-layer spiral microfluidics device with a rectangular cross-section for CTCs isolation from a blood sample [113]; (**e**) Spiral microfluidic with trapezoidal cross-sections for CTCs isolation from WBC [(i–iii) indicate the spiral microfluidic device with an inlet sample and two outlet collections, a cross-sectional view of the channel outlets with the focused particles of different sizes, and experimental separation results under a microscope, respectively.] [97]; (**f**) Bonded multiple layers with a design that consists of four connected spirals [110]; (**g**) Schematic illustration showing cell separation in fishbone units in a multistage microfluidic chip [114] (Reprinted/adapted with permission from Ref. [114]. 2019, Elsevier); (**h**) Triplet-microchannel spiral microfluidic chip with tilted slits for CTC separation [115].

Recently, spiral microfluidic devices have been widely studied by several research groups in the field of particle/cell separation [97,98,109,110]. In two different works, the Papautsky group’s research [102] was inspired to develop a sheathless spiral channel for separating particles of different sizes [95,111]. They proposed 5-loop and 10-loop spiral microchannels with a rectangular cross-section (a width of 100 µm by a height of 50 µm) (Figure 3a). The 5-loop channel was designed to separate the largest and smallest particles with sizes of 7.32 µm and 1.9 µm, respectively, while the 10-loop design was considered to focus 6 µm particles. In addition to validating system performance for cell counting using SH-SY5Y neuroblastoma cells, they claimed a high focusing throughput of 2100 particles per second. Later, the same group designed and fabricated a 5-loop spiral microchannel with a fork-shaped outlet to separate differently sized polystyrene particles (10, 15, and 20 µm) [102]. Like the other inertial platform, the balance between the inertial lift force and Dean drag force was used in their proposed device to focus particles close to the spiral channel’s side walls. They reported 90% and 80% recovery rates for polystyrene particles and neurogenic tumor cells, respectively. Moreover, they claimed that their device had a higher throughput (around 1 million cells per minute) than any commercially available cell sorting technique at the time of publication.

The subsequent research inspired Hou’s group to develop a specific type of spiral microfluidics called Dean flow fractionation (DFF) that continuously separates CTCs from blood samples collected from lung cancer patients [112] (Figure 3b). As shown in Figure 3b, after pumping the blood sample and sheath fluid through the outer and inner inlets, Dean drag forces make smaller hematologic cells (RBCs and leukocytes) follow the Dean vortices toward the inner wall, then return to the outer wall. On the other hand, in addition to Dean drag forces, larger CTCs experience strong inertial lift forces, which force them toward the inner wall of the microchannel. Unlike the other research using blood samples with low hematocrit levels (5%), they used sheath buffer for the first time in a spiral platform to facilitate processing 20–25% hematocrit level blood samples. As a result, using the sheath buffer led to high throughput (3 mL/h) along with resolving the clogging issues and an 85% CTC recovery rate. Later, Hou et al. modified the previous platform for label-free bacteria separation from blood cells with the help of a ribosomal RNA detection method to capture samples with low abundance pathogens from the processed blood sample without culturing or enzymatic amplification [109] (Figure 3c). Although the sensitivity of their modified platform was similar to that of culturing or amplification-involving methods, they could improve the processing time for the bacteria identification test from the whole blood sample by around 8 h compared to culturing or amplification-involving methods. Recently, Warkiani et al. delivered a detailed report on the fabrication and implementation of a multi-layer spiral microfluidics device with a rectangular cross-section for separating CTCs from a blood sample [98] (Figure 3d). They reported a high recovery rate of 85% for CTCs at a relatively high flow rate of 1.5 mL/min using the multiplexed platform.

Guan et al. theoretically and experimentally studied spiral microchannels with rectangular cross-sections with the limitation of low separation resolution, especially when the particles had a close range of sizes [116]. As a result, they developed trapezoidal cross-sectional microchannels to generate a stronger Dean drag force in the outer half of the channel (i.e., the half opposite the center of the spiral channel). This strategy resulted in better separation efficiency due to higher separation distances between particles of different sizes. It was shown that the possibility of clogging and blocking in the channel can be decreased and the detection efficiency can be dramatically boosted if the microchannel cross-section changes from a rectangle to a trapezoid [117]. This shape-changing could reduce the total length of the microchannel and increase the separation rate. Figure 2c illustrates a trapezoidal spiral channel microfluidic chip using inherent Dean vortex flow and inertial lift force to separate head and neck cancer cells by pushing smaller hematologic cells toward the outer wall [106]. Warkiani et al. also proposed trapezoidal spiral microfluidics for ultra-fast and label-free CTC separation [97] (Figure 3e). As can be seen, Figure 3e (i) shows the full spiral design with one inlet and two outlets; (ii) displays a cross-sectional drawing (just before channel outlets) for focusing particles with varied sizes, and (iii) indicates experimental results of the particle separation. They could achieve an 80% recovery rate for different cancer cells (MCF-7, T24, and MDA-MB-231) from 7.5 mL of a blood sample in only 8 min. In a continuation study, Warkiani et al. proposed multiplexed, multi-trapezoid inertial spiral microfluidics with membrane-less microfiltration to resolve the problem of clogging in the membrane filters [110] (Figure 3f). They embedded and integrated forty spiral chips in one setup to increase the flow rate up to 500 mL/min (compared to 6 mL/min for a single chip). The integrated design successfully separated CHO (10–20 µm) and yeast (3–5 µm) cells with a high separation efficiency of 90%. Later, Gao et al. reported 90% separation efficiency with 84.96% purity using hydrodynamic forces in a fishbone-shaped channel microfluidic chip with a rectangular reservoir and inertial focusing microchannel for CTC isolation [114] (Figure 3g).

A couple of spiral channels have been recently integrated to enhance the flow rate at the inlet and improve the final separation speed [98]. In such a combined spiral channel, the fluid will enter from one inlet and leave through different outlets (near the inner and outer walls). Indeed, in the curved channels, $F_{L}$ and a drag force ($F_{D}\propto \;{\rho U}^{2}{d\;D}_{h}/R$, where R is the channel curvature radius) originated from the Dean flow (secondary flow) in the channel cross-section impact particles [118]. The fluid in the horizontal center plane is pulled to the outer wall (due to the influence of centrifugal force and the unbalanced radial pressure gradient), while the fluid at the outer wall flows back along the upper and lower bottom surfaces. As a result, two opposite-direction vortices are formed [72]. Under such a circumstance, the combined action of $F_{L}$ and $F_{D}$ can force larger and smaller particles to accumulate near the inner and outer walls, respectively. Therefore, CTCs (with an average diameter of 20 μm) and RBCs (with an approximate diameter of 8 μm) will be collected at separate exits. It can be concluded that spiral structures with the advantages of precise control, simple structure, and high efficiency are suitable for separating CTCs from the RBCs. Chen et al. used inertial and deformability principles for continuous CTC separation to design and fabricate a triplet parallelizing microchannel in a spiral microfluidic chip interconnected with several tilted slits [115] (Figure 3h). According to inertial and viscous drag forces, cells of different sizes were engineered to attain different equilibrium states inside the microchannel, so the bigger CTCs were placed near the central streamline. The final separation capacity for such a design was 90% at a flow rate of 80 mL/h. Later, Shirai et al. presented a hybrid double-spiral microfluidic chip for separating rare cancer cells from whole blood with the potential to be used in cancer diagnosis, monitoring, and treatment [119]. Their proposed chip consists of a long, thin spiral channel, which is used for cell separation based on size and deformability, and a short, wide spiral channel, used for the capture of rare cells using antibody-functionalized micropillars. The results showed high efficiency and specificity for rare cell enrichment (87% separation efficiency for separating A549 cancer cells). Moreover, the RBC lysis-free design of the chip preserves cell viability and enables downstream analysis of intact cells.

To improve the efficiency of the primarily introduced scheme, numerous attempts have been made. A symmetrically curved channel microfluidic device was designed to continuously isolate CTCs (MDA-MB-231, Jurkat, K562, and HeLa) with high-throughput results (Figure 2d) [107]. At an angle of 280, these cancer cell lines were injected into the curvilinear channel with a continuous flow rate increase in the injection volume. The chip’s viability was reported to be more than 94%. Later, a capillary-inserted microfluidic device was fabricated to separate CTCs under viscoelastic flow (Figure 2e) [108]. Overall, 94% of the MCF-7 cells were separated with a purity of ∼97% from leukocytes at a specific flow rate. The two designed outputs enabled the isolation of relocated 5 and 10 µm diameter particles with ~99% separation efficiency. A self-amplified inertial-focused microfluidic device was later developed to separate different types of CTCs, such as lung cancer cells (A549), breast cancer cells (MCF-7), and cervical cancer cells (HeLa) [120]. The device included a narrow zigzag microchannel connected to expansion sites to facilitate separation based on the size-based approach. The device demonstrated ~80% separation efficiency. A subsequent study designed a microfluidic chip for a high-throughput, label-free, inertial–ferrohydrodynamic CTC isolation [121]. It could successfully establish an inertial–ferrohydrodynamic cell separation chip for separating small CTCs with ~1–2 µm diameter differences in ferrofluids under a magnetic field with a high recovery rate and suitable purity. Recently, Islam et al. designed a microfluidic device for the continuous and label-free separation of CTCs from whole blood using dielectrophoresis (DEP)-based inertial microfluidics in a zigzag channel. [122]. Their device used a combination of inertial lift forces and DEP forces to separate CTCs from RBCs and WBCs based on differences in their physical properties. The zigzag channel design enhanced the separation efficiency by inducing lateral migration of cells, increasing the residence time, and minimizing cell–cell interactions. The device was tested using both artificial and clinical blood samples, and it showed high efficiency and specificity for CTC separation, with a recovery rate of 86.7% and a purity of 91.6%. This means that out of all the CTCs in the sample, 86.7% were successfully captured with the device, while 13.3% were not. The purity of 91.6% indicates that out of all the cells captured with the device, 91.6% were CTCs, while the remaining 8.4% were other blood cells such as RBCs and WBCs. The captured CTCs were also shown to maintain their viability and to be suitable for downstream analysis, such as genetic profiling and drug testing. They also numerically optimized the critical parameters such as the Reynolds number, dimensions of the zigzag channel, voltage, and electrode configuration to improve the separation efficiency.

### 2.4. Inertial Microfluidic Devices with Sudden Changes in Cross-Section

Flat channels in which the cross-section suddenly changes are another noteworthy type of inertial microfluidics. These cross-section changes are often created by the sudden expansion and contraction of the channel in the path, which causes flow deviation (a similar effect to the curvature in the channel) and forms turbulent secondary flows (Dean vortices). The secondary flows in such microchannels efficiently contribute to size-based separation and lateral particle migration [123]. Therefore, particles are initially focused on dynamic equilibrium positions near the sidewalls under the influence of $F_{LW}$ and $F_{LS}$ in the straight channel. When the concentrated particles enter the expansion area, $F_{LS}$ will be the dominant force for particle migration due to the lack of channel walls. Under such a circumstance, when particles are passing through the expansion region, small ones migrate gradually without entering the vortex, while larger ones move laterally into the chamber due to larger $F_{LS}$ and rotate with the vortex. Zhou et al. investigated this phenomenon and studied a microchannel with a similar structure using experimental and numerical simulation approaches [124]. They indicated that particle recirculation within the channel expansion region was dominated by low velocity and drag force, creating particle rotation orbit in the chamber that was connected to particle diameter and flow conditions. Later, Wang et al. added two side outlets to the corners of the chambers and three resistance devices to the outlets in the abovementioned channel structure [125]. Under the optimized experimental conditions, they reached a ~92% purity rate for 21 and 18.5 μm particles. They showed that the flow rates at the outlets could be controlled by adjusting the resistance ratio between the side outlets and the main outlet. After optimizing the microfluidic device performance, small particles were expelled through the main outlet and large particles through the side outlets. In another study, Wang et al. connected another expansion region to the original channel for secondary separation of the ternary mixture [126]. They redesigned the microchannel resistance network to separate particles with three different diameters (21, 18.5, and 15 μm). Although they successfully achieved a 99% separation efficiency for the smallest particles, the separation efficiency for the largest was not satisfactory. Later, they successfully developed an integrated inertial microfluidic vortex sorter for continuous size-based separation of rare cells from diluted human blood with the help of sheath flow with a 90% separation efficiency [127].

**Figure 4:**
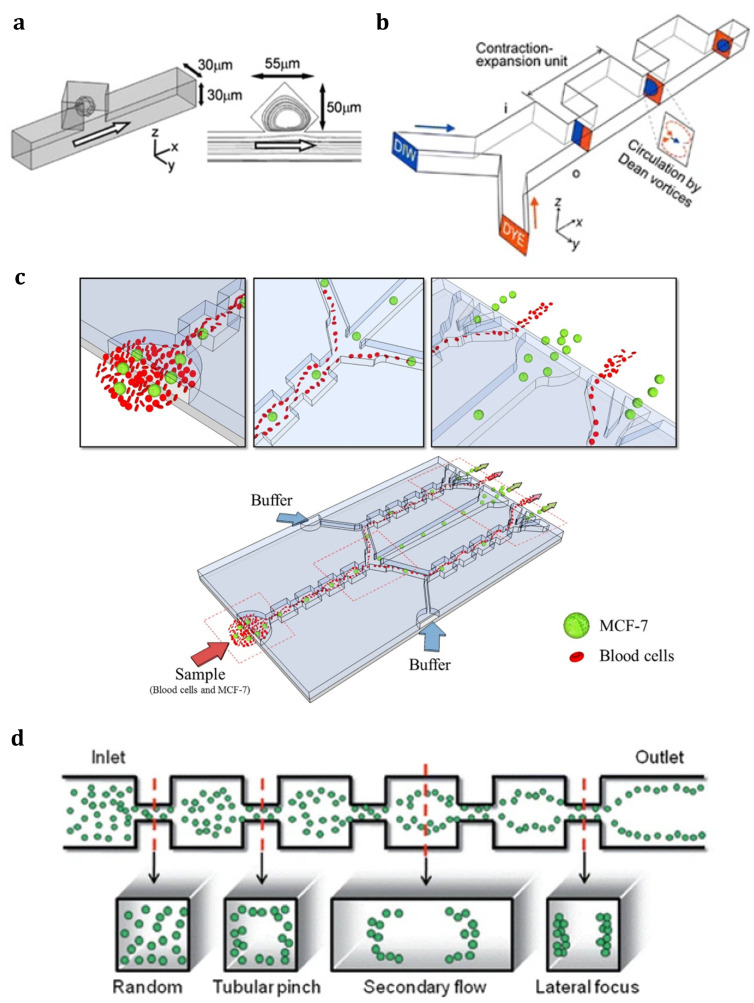

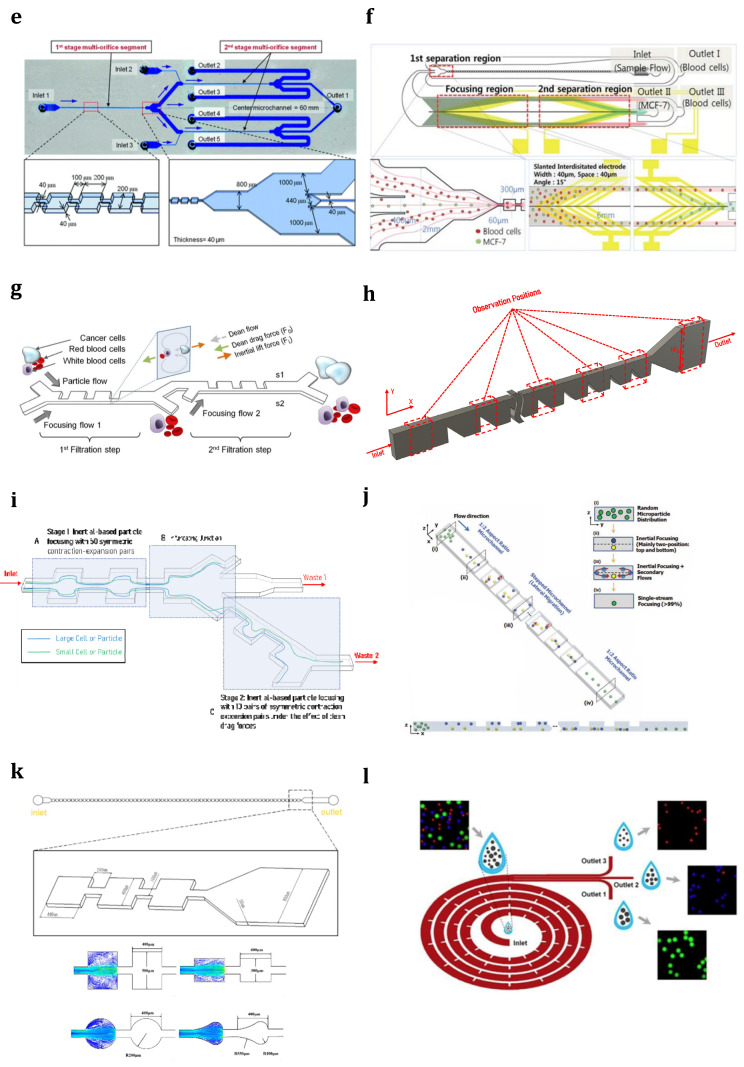
The passive cell separation technique: (**a**) A microfluidic channel with a single microchamber (single vortex) [128] (Reprinted/adapted with permission from Ref. [128]. 2003, Springer Nature); (**b**) A microfluidic channel with several microchambers on one side [130] (Reprinted/adapted with permission from Ref. [130]. 2009, AIP Publishing); (**c**) A multi-orifice flow fraction structure [131] (Reprinted/adapted with permission from Ref. [131]. 2013, AIP Publishing); (**d**) A microfluidic channel with multi-chambers on both sides [132] (Reprinted/adapted with permission from Ref. [132]. 2009, Royal Society of Chemistry); (**e**) Schematic view showing an MS-MOFF device [133] (Reprinted/adapted with permission from Ref. [133]. 2011, Royal Society of Chemistry); (**f**) Schematic view showing the microfluidic device for CTC separation using MOFF and dielectrophoresis (DEP) techniques [134] (Reprinted/adapted with permission from Ref. [134]. 2011, Royal Society of Chemistry); (**g**) Asymmetric CEA microchannel for CTC isolation with two-step filtration [135] (Reprinted/adapted with permission from Ref. [135]. 2013, American Chemical Society); (**h**) Schematic illustration showing a series of abruptly contracted and expanded structures on one side of a microchannel [136]; (**i**) A combination of symmetry and asymmetry CEA channels in a microfluidic device for CTC separation [(A–C) show the first stage of inertial-based particle focusing with 50 symmetric contraction expansion pairs in which particles are focused to the outer walls of the microchannel through a balance of wall lift and shear gradient lift forces, the trifurcating junction, and the second stage of inertial-based particle focusing with 10 pairs of asymmetric contraction expansion pairs in which small particles travel to different equilibrium positions under the effect of dean drag forces, respectively.] [137]; (**j**) A series of contraction regions in a low-aspect-ratio straight microchannel in a single-stream inertial microfluidics [138] (Reprinted/adapted with permission from Ref. [138]. 2013, John Wiley and Sons); (**k**) A schematic diagram showing a CEA channel with different structures and constant contraction–expansion ratios [139] (Reprinted/adapted with permission from Ref. [139]. 2019, Springer Nature); (**l**) The presence of obstacles in a spiral microchannel [140] (Reprinted/adapted with permission from Ref. [140]. 2017, Royal Society of Chemistry).

In addition to using curved microfluidic spiral channels to create secondary flow, microvortices with inertial migration have also been presented with single or multiple cavities/chambers on one or both sides of the microfluidic channels. Shelby et al. developed a straight microchannel (30 µm height by 30 µm width) with the integration of a single diamond-shaped chamber on one side of the channel to generate high radial acceleration microvortices [128] (Figure 4a). The microchamber’s presence led to detaching the fluid flow at the microchamber opening and generating recirculation/vortex flow in the diamond chamber. It was shown that flow velocity could be increased from 3 m/s in the main channel to 12 m/s in the microchamber by optimizing the microchamber dimensions and opening angle. This was the first time a microcirculation/microvortex was used to improve passive liquid mixing in a straight microchannel. What is more, Shelby used red polystyrene beads and green slice beads with two different densities (1.5 and 1.8–2.0 g/cm^3^, respectively) to show the potential applications of a microvortex. The green beads were centrifuged toward the microchamber’s outer edge, while the red beads were focused near the chamber center by increasing the mainstream flow rate from 1.5 to 20 m/s. Based on the same idea, the effect of centrifugation/vortices on different types of cells was studied [129]. The microvotex technique revealed different effects of centrifugation on a single-cell level, including tensile stress (which causes the relocation of intracellular organelles) and shear stress (which causes physical changes in the cell surface) compared to traditional centrifugation approaches.

The design of expansion and contraction arrays can be symmetric, such as combining sequential multi-orifice flow fractionations (Figure 4c) [131], or asymmetric, such as having the arrays at one side of the channel (Figure 4b) [135]. Figure 4b shows a series of microchambers, known as a contraction–expansion array (CEA), integrated on a single side of the microchannel [130]. Numerous theoretical and experimental works have been performed to reach such a design. First, Lee et al. developed a CEA on one microchannel side to achieve laminar mixing between two liquids [130]. It was revealed that their design resulted in Dean-like vortices at the entrance to the contraction region due to the acceleration and deceleration of streamlines. As in any curved/spiral channel, two vortices (an upper counterclockwise vortex and a lower clockwise vortex) were generated, as seen in Figure 4b. The two counter-rotating vortices in this figure drove the deionized water toward the channel center, surrounded by the fluorescein isothiocyanate. Later, the same microfluidic platform was used for several applications, such as three-dimensional hydrodynamic focusing of RBCs [141], inertial separation of differently sized polystyrene beads [142], blood plasma separation [143], and label-free CTC separation from whole blood [135]. Mach et al. developed several CEAs to further improve the CEA platform throughput (in the range of mL/min) by connecting multiple CEA channels in parallel with a single input and a single output [144]. It should be mentioned that they intended to use the microvortices to capture larger particles, not to focus them. When the larger cells/particles approached a microvortex, they were centrifuged near the center of the vortex and remained there, while the smaller particles traveled with the mainstream. Their design successfully captured target particles, which were then fluorescently labeled with a medium exchange process without requiring manual pipetting or washing steps. In this device, the flow rate was decreased to weaken microvortices and release the captured particles/cells into the main flow. This technique successfully separated CTCs and mesothelial cells from the background mixture [144,145].

The multi-orifice flow fractionation (MOFF) design with axisymmetric CEAs on both sides of the microchannel was first introduced in 2008 (Figure 4c,d). Like the single-sided CEA mechanism, a double-sided CEA concentrates cells/particles in a distinct path by balancing the inertial force and microvortices on both sides. As a pioneer in using the double-sided CEA mechanism, Park et al. presented a microchannel with 80 repeated contraction–expansion cycles to continuously separate 7 µm polystyrene divinylbenzene (PS-DVB)) with a high throughput [132]. It was shown that the focusing position was transferred from the channel sides to the channel center due to the increase in the particle Reynolds number ($Re_{p}$) (from the range of 0.8–2.3 to 3.0–3.5). Later, Park used this MOFF platform to isolate differently sized particles (platelet, RBC, and WBC) from the bloodstream [146]. Although a continuous separation process with an intermediate flow rate (1–5 × 10^4^ particles/s) and without the requirement of sheath fluid was achieved using MOFF structures, low purity of 36.4% maximum was shown for 15 µm particles. Moreover, although enlarging the collection region led to an increase in the recovery rate, it dropped the level of separation purity to a low of 15.5%. As a solution for system performance improvement, they proposed having a specific flow rate for separating each type of particle from the background mixture.

The Park group’s research inspired generations of future researchers. Sim et al. recently developed a design improvement to the MOFF platform, called a multi-stage–multi-orifice flow fractionation (MS-MOFF) device by connecting two sets of multi-orifice segments at the outlet of the main channel to improve the recovery rate [133] (Figure 4e). Indeed, their design consisted of one CAE in the first stage and two CAEs in the second stage. In this mechanism, CTCs were firstly focused in the channel center and then moved to the middle of the channel outlet, while the blood cells and a few unfocused CTCs settled near the two sidewalls and were directed to the side channels. The unfocused MCF-7 cells were then isolated to the channel center. The MS-MOFF structure was reported to achieve a higher recovery rate (88.5%) than a typical MOFF platform for a 15 µm particle size. At the same time, with the help of the Park group’s findings, Moon et al. improved the recovery rate of MOFF by combining MOFF with the dielectrophoresis (DEP) method [134] (Figure 4f). Their integrated platform could separate and enrich up to 162-fold further MCF-7 from a blood sample at a flow rate of 126 µL/min. Later, another microfluidic chip was proposed by connecting four parallel MOFFs to improve the final separation efficiency [131]. Recently, Bakhshi et al. designed an integrated microfluidic chip for two-embedded stages, label-free, and continuous CTC separation from blood cells with the help of hydrodynamic inertial focusing and the dielectrophoresis (DEP) separation method [147]. In their numerical simulations, they investigated the effects of the aspect ratio, dielectrophoretic force, channel size, flow rate, separation efficiency, and shape on CTC separation efficiency. Their proposed design numerically yields viable CTCs with 99.5% isolation efficiency.

The asymmetric channel has a more diverse structure and flexible application than the symmetric channel. At first, Lee et al. developed a three-dimensional hydrodynamic focusing using an asymmetric contraction–expansion microchannel with a single sheath flow to separate MCF-7 from whole blood and achieved a recovery rate greater than 99% [135] (Figure 4g). After the fluid entered the contraction region from the expansion region, the sample flow was forced to the sidewall by the centrifugal effect. Indeed, the centrifugal effect induced a secondary flow to make the sheath flow envelope the sample flow and focus it on the cross-section. Fan et al. designed and fabricated an asymmetric contraction–expansion microchannel with a series of asymmetrical sharp corners on one side of the channel (Figure 4h) and achieved the single-stream focusing of particles with varied sizes (7.32, 9.94, and 15.45 μm) over an extensive range of Re (from 19.1 to 142.9) [136]. These sharp corners induced centrifugal force, which caused the particles to migrate to the opposite sidewall without sharp corners for single-stream focusing of microscaled particles under a specific flow rate range. In another study, based on their previous results, they examined continuous 3D particle focusing by modifying two sides of the microchannel (to have symmetric sharp corner structures) and combining a 90° curved channel [148]. Indeed, the Dean flow-induced drag force first made the particles focus at the center plane in the curved channel, and then the effects of the symmetric sharp corner structures were responsible for focusing them at the channel center. Later, Yang et al. combined symmetry and asymmetry contraction–expansion channels to develop a novel microfluidic device for the label-free separation of CTCs [137] (Figure 4i). In the first stage, the symmetry channel was responsible for focusing all particles into tight streamlines close to the two sidewalls. In the second stage, the asymmetry channels sorted particles of different sizes. Chung et al. also combined the inertial effect and secondary flow by inserting a series of contraction regions in a low-aspect-ratio straight microchannel to achieve an inertial microfluidic single-stream particle focusing [138] (Figure 4j). Randomly distributed particles were first focused toward two equilibrium positions through the upstream straight channel. The focused particles were then relocated due to the secondary flow induced by the stepped channels. According to these findings, the original contraction sections were replaced with expansion regions to develop a new structure in the stepped microchannel for single-line focusing of cells and for studying Re and AR effects on the focusing and rotation of cells with different aspect ratios and sizes [149].

In addition, Sollier et al. arranged multiple expansion–contraction chambers (in series and parallel) to develop a microfluidic device with channel throughput improvement for processing large-volume samples [145]. Their group also examined the effects of the channel aspect ratio, channel length, blood dilution, and throughput on particle sorting. Later, Dhar et al. used Sollier et al.’s research to investigate channel cross-sectional geometry and its effects on separation efficiency and capture stability [150]. They showed that the channel cross-sectional region played an essential role in the threshold size of the captured particles. Meanwhile, in another study, the original long upstream straight channel was superseded with 1000 μm spaced contraction–expansion chambers, which resulted in a 1.6 times higher capture efficiency of MCF-7 [151]. Subsequently, a system integrated with the vortex trapping and deformability cytometry (VDC) technique was proposed to seamlessly capture, release, and measure rare target cells using the above-mentioned modified microchannel structure [152]. In addition, the vortex chip was revised by adding lateral and connection channels to the expansion–contraction chamber and investigating the effect of their different dimensions on the capture efficiency improvement in the chip [153]. Recently, Liu et al. revealed that the circular channel had the best CTC separation performance among four sets of contraction–expansion microchannels with different structures and shapes and constant contraction–expansion ratios [139] (Figure 4k). Later, it was shown that reasonably increasing the contraction–expansion ratio could provide a relatively high throughput separation of particles with different sizes [154]. For the channel with a large contraction–expansion ratio, it was also found that a higher flow rate was required to focus particles with varied sizes at their respective equilibrium positions (large particles in the channel center, small particles near the two sidewalls), and small particles were more easily driven away from the center. The damage to and loss of cells during the contraction–expansion and spiral microchannel sorting process were studied [155]. The results showed higher deformability of cells in the spiral channel than in the contraction–expansion channel. In other words, there was a higher possibility of damaging the intracellular structures of cells while passing the contraction–expansion channel. Recently, Islam et al. introduced a DEP-based contraction–expansion inertial microfluidic channel for CTC separation [156]. They combined curved contraction–expansion channels with integrated electrodes for DEP manipulation of cells and inertial separation methods to separate CTCs from WBCs regardless of the size overlap. The channel design incorporates a series of contraction–expansion regions that generate Dean vortices, which create strong inertial lift forces that selectively separate A549 CTCs from WBCs based on differences in size and deformability. The integrated DEP electrodes further enhance the separation efficiency by selectively capturing and repelling cells based on their electrical properties. Furthermore, the proposed method allows users to modify the cell migration characteristics by controlling the number of contraction–expansion sections in the channel, the flow rate, and the applied voltage and frequency.

Contraction–expansion microchannels can be designed in a curved [157], spiral [140,158], or serpentine [159] channel pattern or combined with obstacles [160] to enhance separation performance and improve the manipulation performance. For example, a three-stage straight microchannel with different structures, including a rectangular expansion channel with a series of cylindrical obstacles, a square channel (AR = 1.0) for its entrance, and a rectangular channel (AR = 2.0) for its exit, was developed for single-stream particle focusing [160]. In this device, the particles were first settled in four equilibrium positions on the square channel, then the pillar-induced secondary flow forced them to migrate into a narrow band, and finally, the wall-induced inertial lift force dominated them for single-stream focusing in the high aspect ratio channel. To explore particle separation based on the combination of contraction–expansion structures and spiral or serpentine microchannels, Shen et al. developed a fast, high throughput, and high-efficiency particle focuser and sorter by adding obstacles to the spiral microchannel [140] (Figure 4l). The obstacles significantly accelerated the secondary flow on the cross-section, which is advantageous for particle focusing.

Furthermore, a spiral microchannel merged with contraction–expansion structures was proposed [158]. This design tried to fix the equilibrium position of the particles in the conventional spiral microchannels under different channel dimensions and flow rates. Generally, asymmetric contraction–expansion channels expand particle manipulation choices. This means that the asymmetric contraction–expansion channels provide significant benefits for particle single-line focusing and overcome the disadvantages of symmetric inertial contraction–expansion channels. For example, non-orthogonal contraction–expansion arrays can manipulate particles over a wide range of Re. In addition, the asymmetric structures may be adapted to connect with other microchannels to make particle manipulation more stable, efficient, and sensitive.

### 2.5. Advantages and Limitations of Inertial Microfluidic Devices for Particle Separation Applications

Compared to the other separators, the advantages of inertial microfluidic devices include (1) proficiency, fabrication, and operation, (2) high-throughput and continuous sample processing, (3) neither barriers nor mechanical or electrical components are required in their structure, (4) they can be quickly and easily fabricated using different microfabrication techniques, especially PDMS softlithography, and (5) the whole system can be run with only one syringe pump.

On the other hand, some weaknesses of the inertial microfluidic method are listed below. First, the maximum amount of inertial lift force in inertial microfluidics can be obtained if the sample only includes rigid spherical particles. Second, adequate inter-particular distances can only be reached if the concentration of particles is sufficiently low. This is because a high concentration of particles can affect hydrodynamic focusing. Third, a suitable focus is only achieved if the flow rate is maintained within a specific range [161]. Last, the inability to accurately control fluid flow in an inertial microfluidic system is another problem [161]. A self-controller might be a good candidate for regulating the fluid flow in these devices. Still, installation difficulty and the application of such controllers have not been studied for separation purposes in these microfluidic devices. Although soft self-controllers with embedded instructions have been recently proposed [162,163], their efficiency has not yet been evaluated in connection with the other components and units. A common concern associated with installing the self-controller on a multilayer microfluidic device is the leakage problem, which has not been thoroughly addressed. As a result, a kind of flow control mechanism for inertial systems is still in demand. This limitation in regulating the fluid flow has led researchers toward centrifugal microfluidics, which does not require such equipment.

## 3. Centrifugal Microfluidics

### 3.1. Introduction

The emergence of the lab-on-a-chip (LOC) concept in the field of microfluidics is considered the turning point of achievement. Due to the development of LOC technology, chemical and biological experiments can be performed on an all-in-one, automatic LOC device, which demands less reagent, a smaller sample volume, and a shorter processing time. Indeed, a set of microchannels, micropumps, microvalves, micromixers, and other components, usually accessible in a macroscale lab, are established in such a device to perform and analyze experiments. For example, if we assume the microchannel dimensions of about one-thousandth of a laboratory tube, the required material for testing will be reduced by about one million times. While a macro-dimension experiment consumes one liter of raw material, only one microliter of the sample is approximately spent in microsystems. This dramatic reduction minimizes both the amount of test material and the material supposed to be added to the sample for testing. The livability and biodegradability of the applied material in these inexpensive systems make them disposable, which is an advantage for blood tests as it reduces contamination. The possibility of infection would affect the test results [22]. The first LOC analysis system was first used in 1979 to investigate gas chromatography applications [164]. Since then, these devices have been investigated for numerous applications, including biosensors [165], separation [166], analysis [167], drug delivery [168], optoelectronics [169], cell manipulation [170], and chemical synthesis [171,172].

### 3.2. Centrifugal Microfluidic Systems in Lab-on-a-Chip Technology

**Figure 5:**
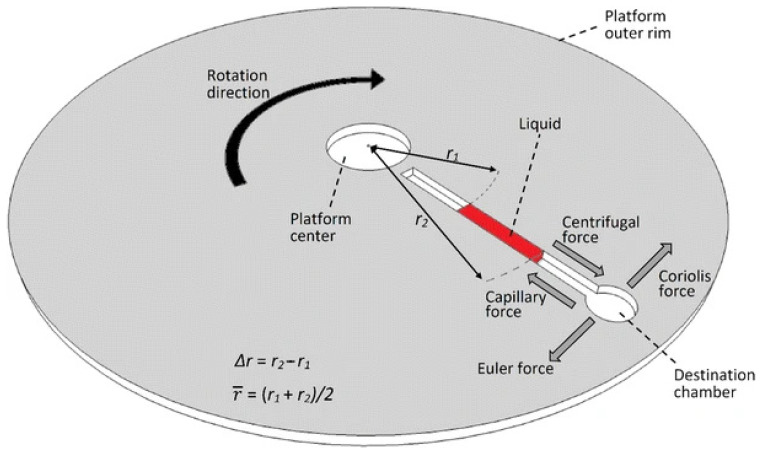
A simple centrifugal microfluidic system with a straight microchannel [91] (Reprinted/adapted with permission from Ref. [91]. 2017, Springer Nature).

The centrifugal microfluidic system, the so-called lab-on-a-CD (LOCD), was developed with inspiration from LOC technologies. A number of microchannels, reservoirs, and other microfluidic components are integrated into a compact disk (CD), which is supposed to rotate at a specific angular velocity. As a main branch of LOC technology, LOCD uses inherent force (as the driving mechanism) instead of classic mechanical or electrokinetic pumping. Figure 5 shows a centrifugal microfluidic platform with a straight microfluidic channel preloaded with a volume of liquid [91]. As can be seen, the principal forces operating on the liquid sample are centrifugal force (pushing the liquid toward the platform’s outer edge), capillary force (acting against the direction of liquid flow), Coriolis force (perpendicular to the liquid flow, and opposite to the rotation direction), and Euler force (perpendicular to the CD rotation direction). Therefore, these driving forces on a rotating platform can control the fluid flow inside the channels without the need for any syringe pump or other external equipment. Furthermore, the number of centrifugal forces can be simply controlled by changing the angular velocity of the rotating system. Moreover, the velocities and effective forces on the fluid are dependent on the second power of the angular frequency. Therefore, changing the rotational velocity can provide an extensive range in the force required for driving or stopping the fluid flow. Centrifugal force can be adjusted to be strong enough to overcome surface tension and capillary forces and cause fluid flow. Furthermore, under the effect of channel geometry, the centrifugal force can reach a minimum value where other forces dominate it and stop the fluid flow. Specifically, the system can be easily controlled and operated [173]. Another advantage of LOCD is having centrifugal force independence of parameters such as viscosity, conductivity, pH, and surface tension.

### 3.3. Applications of Centrifugal Microfluidics in LOCD Platforms

**Figure 6:**
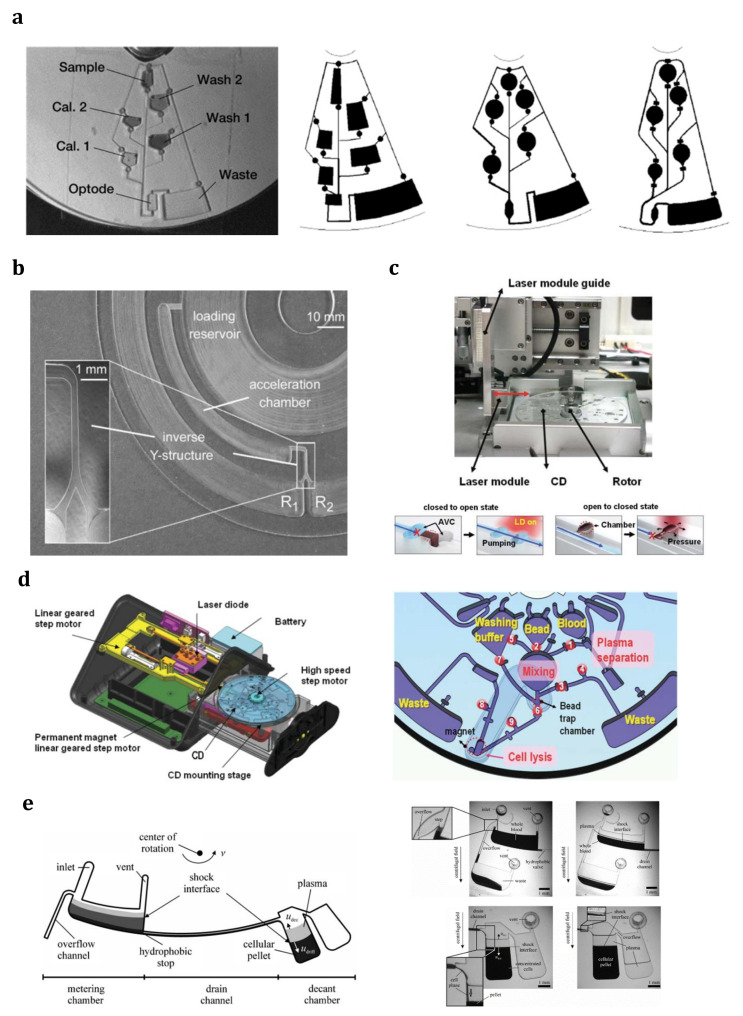
LOCD applications for performing different microfluidic functions: (**a**) Flow sequencing using centrifugal propulsion and capillary valving in a polymer-based CD platform along with different designs for calibration purposes [181] (Reprinted/adapted with permission from Ref. [181]. 2001, Springer Nature); (**b**) The novel flow switch for centrifugal microfluidic platforms controlled with the Coriolis force [184] (Reprinted/adapted with permission from Ref. [184]. 2005, Royal Society of Chemistry); (**c**) A novel microvalve actuated using laser irradiation [185] (Reprinted/adapted with permission from Ref. [185]. 2007, Royal Society of Chemistry); (**d**) Schematic diagram showing the inside of the portable LOCD for DNA extraction from whole blood along with a detailed microfluidic layout and functions [186] (Reprinted/adapted with permission from Ref. [186]. 2007, Royal Society of Chemistry); (**e**) Centrifugal extraction of plasma from sediment with a decanting structure and metering the plasma in consecutive processes using a rotating disk [190] (Reprinted/adapted with permission from Ref. [190]. 2006, Royal Society of Chemistry).

Although the principles of centrifugal analyzer systems were established in the late 1960s, their implementation in microfluidic domains was first carried out by a few commercial organizations in the early 1990s [174]. Following the activities of these pioneering commercial companies, research and academic groups have developed numerous microfluidic processes, including volume measurement [175], gating [173,176,177,178], mixing [179], flow orientation [180], pumping various fluids [173,176], and so on, and mounted them onto centrifuge-based platforms and a CD. As a novel technology for diagnosis and separation, LOCD can combine and perform different microfluidic functions, from mixing and valving to cell separation. Madou et al. established a polymer-based CD platform with different functions and capabilities, such as capillary valves and metering, mixing, and flow sequencing [181]. As shown in Figure 6a, the pre-loaded sample flows in a specific radial path by changing the angular velocity of the platform. It was found that the shape and relative location of reservoirs and channels play significant roles in designing the calibration system in a microfluidic device. It was also shown that sharp corners or turns lead to high residual stress during the fabrication process; therefore, they must be avoided. Later, Riegger et al. used this phenomenon and presented a novel method to determine blood hematocrit using a visual inspection [182]. After that, the first step toward nucleic acid analysis was performed by realizing a purely mechanical cell lysis method using a CD platform [183]. Around the same period, a state-of-the-art controllable flow switch handled using rotation frequency through the Coriolis force was developed on a CD platform by Brenner et al. [184] (Figure 6b). They used the Coriolis pseudo-force to provide a flow control mechanism, which divided an incoming stream between two symmetric outlets in a rotating disk. Their simulations and experiments indicated that the frequency threshold, as a function of the channel geometry, plays a key role in diverting the flow into the desired outlet, so that above a certain threshold frequency, the Coriolis force will be dominant. Under the effect of this dominant force and according to the direction of rotation, the entire flow would enter one of the outlets. Park et al. showed an interesting application of centrifugal microfluidics by presenting a microvalve (the so-called laser-irradiated ferrowax microvalves (LIFM)) that worked based on a phase change and was controlled with laser irradiation [185] (Figure 6c). The nanocomposite materials in the microvalve consisted of iron oxide nanoparticles, which were dispersed in paraffin wax. Due to the strong absorption of laser light by iron oxide nanoparticles, although a very intense laser beam cannot melt wax alone, a relatively weak intensity laser beam can easily melt paraffin wax containing magnetic beads (the so-called ferrowax). Indeed, after being excited with laser irradiation, the heated magnetic beads will transfer the thermal energy to the paraffin wax. The open and close functions in their design were achieved by focusing the laser beam at the location of the microvalve (for the opening purpose) or the pre-loaded ferrowax chamber (for the closing purpose) so that the molten ferrowax flows into a specific chamber (AVC) to open the channel or into the main microchannel to block the channel. In another application, Cho et al. extracted DNA from whole patient blood using a fully integrated LOCD platform [186] (Figure 6d). They proposed a target-specific cell separation and laser-irradiated magnetic bead system (TS-LIMBS) to combine the LIFM control technique, which worked based on the phase transition of ferrowax, with a rapid cell lysis method, which worked with the help of laser irradiation on magnetic particles. Indeed, the laser beam in their application was used for both LIFM control purposes and the cell lysis process.

### 3.4. Applications of LOCD in Chemistry and Biotechnology

LOCD could easily be used for numerous applications in the chemistry and biotechnology fields [185,186]. As a density-based approach, LOCD is used for sample transportation and separation [187], such as plasma separation from blood cells [188] and the extraction of leukocytes from blood samples [189], and, additionally, to separate immune cells [76] and isolate CTCs from whole blood [78]. The outstanding point about LOCD is that different functions, such as mixing, valving, and cell separation, can be incorporated into one microfluidic platform, which, in turn, makes the LOCD a promising technology for diagnostics and point-of-care applications.

### 3.5. Advantages of Centrifugal Microfluidic Systems for Cell Manipulation

As opposed to all inertial microfluidic systems, which must be established on a stable/fixed substrate, this type of microfluidic system is capable of working on both stable and rotational platforms. When an inertial microfluidic system is implemented on a rotating platform, the fluid flow inside its microchannel can be handled with the aforementioned forces [191]. These systems can substantially create and conduct fluid flows with different physical properties, especially for biological liquids and laboratory reagents. Indeed, the centrifugal platform has many beneficial advantages, especially for biological sample handling and cell manipulation [192]. Here are some of these advantages that relate to cell manipulation:

- Using the centrifugation process to execute sedimentation for sample separation and cell enrichment (concentration), which is a straightforward method.
- Independence of centrifugation pumping from the sample parameters (i.e., viscosity, surface tension, electrical conductivity, and pH), which makes it excellent for operating on biological samples such as blood [173,193].
- Reducing sample contamination and enabling the device to be disposable due to the clear separation between the microfluidic system and the pumping/detection components [173,192,193].
- Not using external mechanical equipment to regulate the fluid flow, removing the required instrumentation in contact with external hardware, and eliminating the syringe pumps. An ordinary, inexpensive rotary motor generates the rotational motion.
- Removing any disturbing bubbles or residual volume, which is a tremendous achievement over conventional microfluidic systems.
- Regulating the centrifugal forces by changing the drive engine’s angular velocity (because the centrifugal forces (due to the system rotation) link directly to the drive system’s rotational frequency).
- Removing any generated vibrations in the fluid from the pumping methods using pressure differences and self-stabilizing the rotational motion of the disk.
- Having the inherent feature of sample transportation and density-based separation [187].

In addition to the pumping capability of centrifugal microfluidic devices, the inherent centrifugal force in such devices can also separate different components or phases based on their mass density differences. Many passive particle/cell isolation techniques have been reported on the centrifugal microfluidic platform. Several of these reports submitted centrifugal microfluidic platforms as an alternative to conventional centrifugation devices to separate blood components [194,195,196,197]. These various techniques are categorized into:

1. Density-based blood fractionation (sedimentation);
2. Cell/particle separation based on physical properties;
3. Separation based on immunoaffinity processes.

The main motivations behind the high number of research papers and theses in this area are the use of blood as the biological sample for most diagnostic tests and the requirement for portable devices with high throughput to handle blood samples. Recent studies show the importance of their applications in separating blood components, such as CTC [78], plasma [188], immune cell [76], and leukocyte [189] separation from blood samples. For example, the centrifugal force field can manipulate, partition, and separate cells of different sizes and densities from the whole blood sample. Haeberle et al. used this centrifugation-based method to perform on-disk raw blood separation and plasma extraction using a decanting structure in which overflowed plasma emptied out [190] (Figure 6e). Their design consists of a metering chamber, which is connected to two subsequent chambers through a drain channel. The metering chamber was used for sedimenting and sustaining the cells. The metering of a raw blood sample to a specific volume was performed using an overflow channel (located next to the inlet) and a hydrophobic stop (located at the outlet of the metering chamber). After that, the metered sample went toward the decant chamber via the drain channel. The subsequent reservoir received the purified plasma via a decanting mechanism. Their continuous centrifugal flow separation technique could extract 2 µL plasma from a 5 µL raw blood sample in 20 s using moderate rotating frequencies of 40 Hz. Remarkably, one of the most studied blood separation techniques in centrifugal microfluidic systems is separating the plasma component from the rest of the blood cells [198]. According to research in this field, the plasma separation procedure consists of two main steps: first, cell sedimentation using centrifugation, and second, plasma extraction from the layer-separated sample [198].

### 3.6. Density-Based Blood Fractionation (Sedimentation) in Centrifugal Microfluidic Platforms

**Figure 7:**
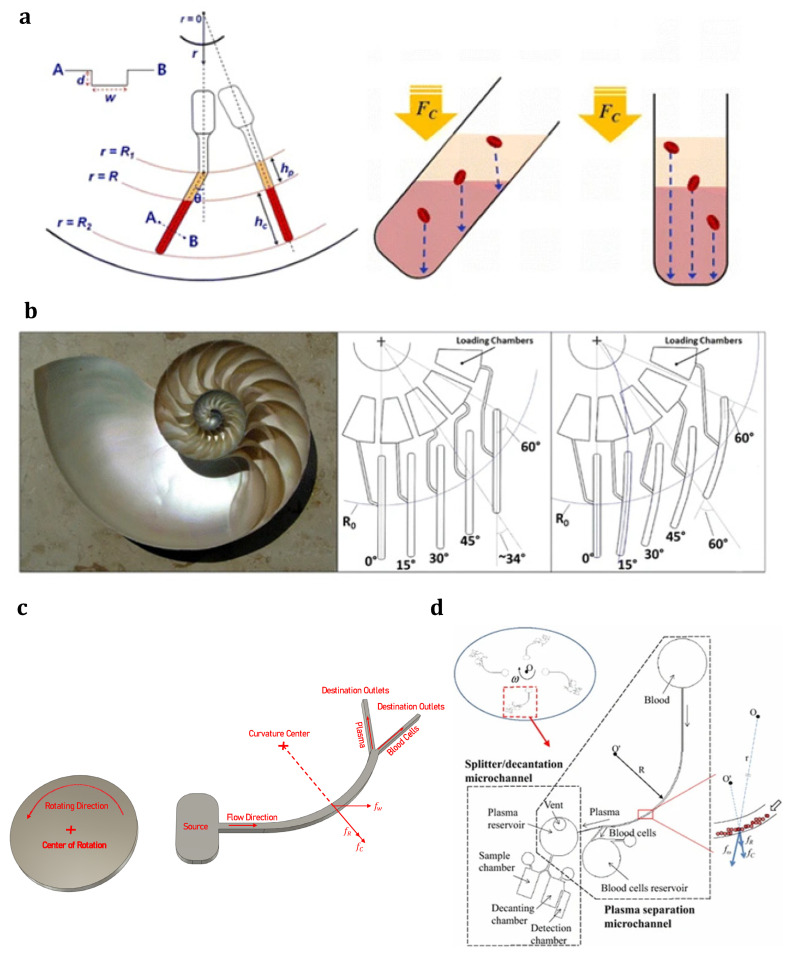
Centrifugal microfluidic device as a passive cell separation technique: (**a**) Tilted sedimentation chamber effect on plasma separation based on the centrifugation/sedimentation process [195] (Reprinted/adapted with permission from Ref. [195]. 2013, Elsevier); (**b**) The spiral mirabilis sedimentation chamber effect on plasma separation based on the centrifugation/sedimentation process [196] (Reprinted/adapted with permission from Ref. [196]. 2013, Elsevier); (**c**) Blood fractionation on a centrifugal microfluidic platform with a curved channel to separate blood plasma from the other components in the blood [194]; (**d**) Schematic illustration showing microchannel networks on the proposed centrifugal microfluidic disk for blood assays [202] (Reprinted/adapted with permission from Ref. [202]. 2015, Springer Nature).

The plasma separation step is usually accomplished in a sedimentation chamber or another radially arranged microstructure on the centrifugal microfluidic platform [192,197]. During centrifugation, the sedimentation chamber structure pushes the higher density of the blood portion (blood cells) to settle at the bottom of the chamber, while the lighter portion (i.e., plasma) remains at the top of the blood cell layer. The influence of the sedimentation chamber geometry on the plasma separation purity and processing time was studied in [195,196]. It was found that the separation process could be accelerated by up to 8-fold after narrowing the sedimentation channel and increasing the tilt angle of that channel with respect to the radial direction. This was similar to Boycott’s discovery in 1920 when he explained that particle sedimentation would occur faster in slanted chambers due to the larger accessible surface area (the side wall and base of the chamber) for settling particles [199]. Another reason for the acceleration in the sedimentation process is the shorter distance the particles/cells migrate to reach the chamber wall than the chamber base. This is similar in narrower chambers where the side walls are closer together [199]. The difference between straight and tilted sedimentation channels is shown in Figure 7a [195].

Figure 7b presents a curved design of the sedimentation chambers in a LOCD with a logarithmic spiral or mirabilis design [196]. Such a spiral design increased the separation process speed by 39% compared to straight conventional sedimentation chambers because it provides more surface area for sedimentation (similar to a tilted chamber) and has a constant centrifugal force (applied along the entire channel length compared to the variable centrifugal force applied along tilted chambers). Later, another study discussed how cells might diffuse into the already separated plasma after centrifugal based-separation, especially at the end of the centrifuge process when the rotation is stopped [200]. As a solution, the researchers introduced out-of-plane microvalves and triangular obstacle structures (TOSs) to tackle this problem. Moreover, a particle size-independent passive method using reserved pneumatic energy was proposed for particles/liquid sedimentation, resuspension, extraction, and transport on a centrifugal microfluidic platform without using any external force or special coating of the microfluidic structure and just by controlling the platform rotational speed [201].

After completing the sedimentation process, decantation or extraction of purified plasma and the following steps are performed. The extraction step is usually achieved using a siphon channel [198] or a straight channel controlled with a valve [197]. The intersection of the extraction channel and the sedimentation chamber lies slightly above the interface between the plasma and RBC layers. The siphon channel can be passively actuated compared to a straight channel where an active valve is required. When the platform’s rotational speed is decreased, the siphon channel’s hydrophilic property pulls the plasma into the channel through the crest. Increasing the spinning speed will drive the plasma into the collection chamber.

Zhang et al. used a different plasma/cell separation mechanism and developed a simple microfluidic system for isolating blood plasma from the other components [194]. Figure 7c shows their design, which consists of a short straight microchannel leading to a curved microchannel, followed by two collection reservoirs (one for plasma and the other for RBCs). As can be seen from this figure, the effect of centrifugal force ($f_{w}$) and the Coriolis force ($f_{c}$), and the secondary centrifugal force ($f_{R}$) (due to the curvature structure of the microchannel), helps to separate blood components. They also explained the degree to which these three forces contribute to the separation process. As the blood sample moves through the microfluidic networks, plasma flows closer to the microchannel’s inner wall (which is nearer to the CD center) due to the lower density of plasma, while the blood cells are pushed to migrate closer to the microchannel’s outer wall (which is nearer to the CD edge) due to the higher density of blood cells. Finally, when reaching the Y-junction of the channel, two parallel streams of plasma and blood cells flow toward the embedded collection chambers and are separated. Although the authors claimed a 90% separation efficiency for a 5% hematocrit level blood sample, the separation efficiency decreased to 65% when processing whole blood (with 48% hematocrit).

Based on the same fundamental concepts, an extended secondary microfluidic device in a situation of having a mixture of the extracted plasma and a specific reagent was developed in [202] (Figure 7d). The authors used siphoning and mixing structures to perform prothrombin time (PT) tests and implemented a slightly different design with a decanting chamber to perform creatinine tests. They claimed a dramatically faster process with a 96% high separation efficiency and a short processing time of 5–6 s compared to conventional techniques. As a conclusion for extracting plasma from the layered blood sample, the curved channel method does not require a complex microfluidic setup with siphon channels compared to the earlier-described sedimentation methods. According to these studies, passive plasma/cell sedimentation plays a vital role in recognizing the centrifugal microfluidic platforms for fully integrating biological multi-step assays in an automated fashion without needing external interaction from a highly expert clinical technician.

### 3.7. Cell/Particle Separation Based on Physical Properties in Centrifugal Microfluidic Platforms

**Figure 8:**
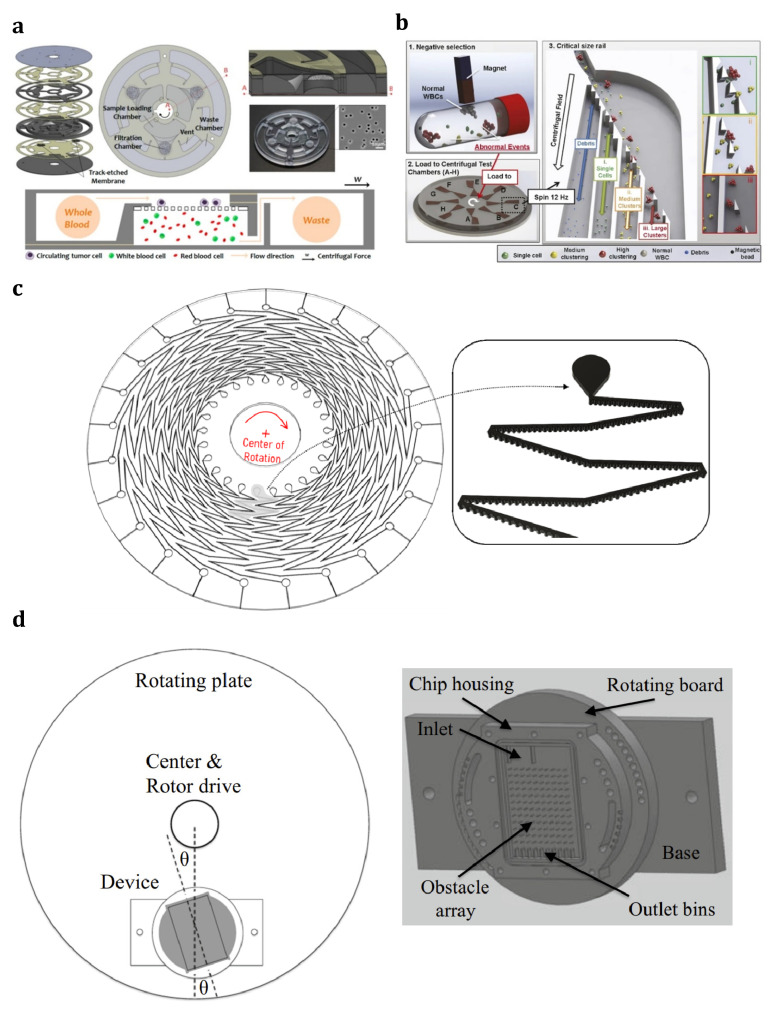

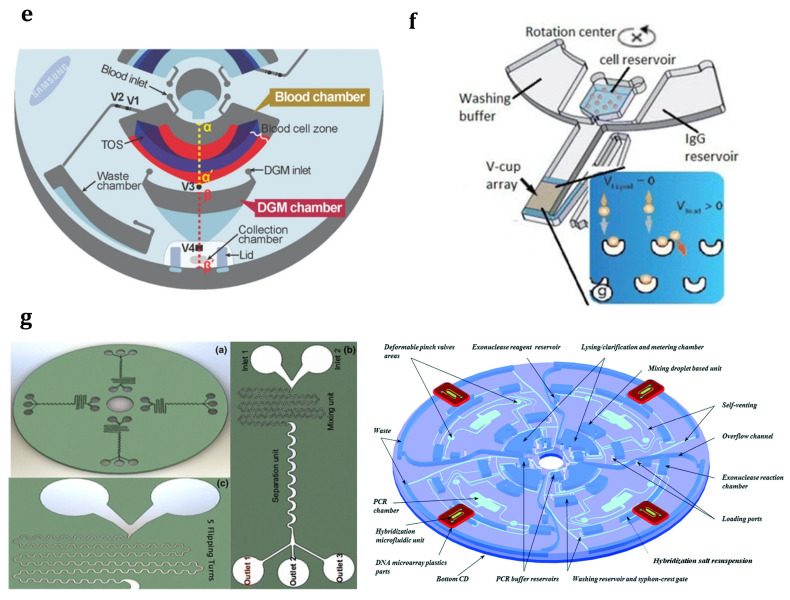
Centrifugal microfluidic systems on a disk: (**a**) Cell separation using a membrane filter; the microfluidic CD contains three sets, and each set includes a loading chamber, filtration chamber, and waste chamber [78] (Reprinted/adapted with permission from Ref. [78]. 2014, American Chemical Society); (**b**) A rail of gaps with increasing opening size for measuring the size distribution of CTCs clusters [203]; (**c**) A centrifugal microfluidic disk with zig-zag-shaped microchannels for single cell isolation [205]; (**d**) A schematic view showing a centrifuge-based deterministic lateral displacement (CDLD) separation system [206] (Reprinted/adapted with permission from Ref. [206]. 2016, Springer Nature); (**e**) A microfluidic design consisting of a blood chamber, DGM chamber, collection chamber, and waste chamber for CTC isolation from a blood sample [197] (Reprinted/adapted with permission from Ref. [197]. 2014, American Chemical Society); (**f**) A microfluidic design with a V-shaped trap array consisting of a cell reservoir, washing buffer reservoir, IgG reservoir, V-cup array, and waste chamber [207] (Reprinted/adapted with permission from Ref. [207]. 2012, Royal Society of Chemistry); (**g**) A sketch showing a centrifugal microfluidic system with the integration of a micromixer and inertial separator units for immunoaffinity-based CTC separation [208,209] (Reprinted/adapted with permission from Ref. [208]. 2015, Royal Society of Chemistry) & (Reprinted/adapted with permission from Ref. [209]. 2015, Springer Nature).

Previously, different microfluidic methods for separating cells in general from blood plasma were presented. Cell/particle separation based on physical properties describes isolation methods for extracting certain cell/particle types from a relevant background population. Typically, these methods are used to enrich targeted cell types while discarding undesirable cells. Geometry-based cell/particle separation mechanisms in centrifugal microfluidic systems have been reported in numerous studies [78,203,204,205,206]. Lee et al. developed a centrifugal platform with three processing sets (each set contains a sample loading chamber, filtering chamber, and waste chamber) to separate CTCs [78] (Figure 8a). This device integrated a membrane filter (with a pore size of 8 µm) into the filtration chamber to trap the target CTCs. Although the processing time to filtrate 3 mL of the sample took only 20 s, the postprocessing, including washing, blocking, incubation, staining, and cell analysis, took around 50 min. Moreover, although they reported a high count of captured WBCs (3092 cells) and a high CTC capture efficiency (84%) at a relatively low rotational speed (600 rpm), the capture efficiency and WBC count dropped to 50% and 181 cells, respectively, at a high spinning speed of 3600 rpm. The authors compared their device with the commercial microfluidic platform for CTC isolation (ScreenCell^®^) to experimentally verify their device. After the comparison, their introduced platform and ScreenCell^®^ system reported capture efficiencies of 56 and 69%, respectively. They claimed that the slightly higher efficiency of the commercial separator originated from the smaller pore size in the used membrane filter (7.5 µm) and the use of dilution FC_2_ buffer to stabilize the cells. Their proposed microfluidic platform was the only experimentally validated system compared to a commercially approved CTC separation approach.

Glynn et al. implemented a radially inclined rail featuring a series of gaps with increasing opening size to separate different size variation-based clusters before sending them to different destination chambers [203] (Figure 8b). They developed a novel two-stage, stopped-flow, continuous centrifugal design to measure the size distributions of CTC clusters in a blood sample. Later, the counterflow centrifugal elutriation (CCE) technique was implemented on a centrifugal microfluidic system for size-based particle sorting [204]. The method relied on balancing the centrifugal and liquid drag forces, which pushed particles toward the platform edge and center, respectively. Bigger particles flowed near the chamber inlet because the net force was higher near that area, while smaller particles moved toward the chamber outlet due to the lower net force near the chamber outlet. Their device could successfully separate differently sized polymer particles (1, 3, and 5 µm in diameter) and blood cells (erythrocytes and leukocytes) from the diluted blood sample.

Kubo et al. developed a zig-zag-shaped microfluidic device with a total of 24 microchannels from the center to the outer edge and 530 U-shaped capturing chambers placed along the sides of the channels on centrifugal platforms to reach single-cell level analysis [205] (Figure 8c). They investigated the possibility of entrapping a single cell from a cell suspension in microchambers engraved on a rotating platform. The processing time for isolating various cell types (Escherichia coli, baker’s yeast, Jurkat cells, and NIH3T3) was 30 s at 3000 rpm. Later, Jiang et al. developed a centrifugal deterministic lateral displacement (CDLD) separation system by integrating a square array of cylindrical posts at specific tilting angles with respect to the direction of centrifugal force to passively separate particles [206] (Figure 8d). They examined the effect of different tilting angles on the migration of spherical particles with different sizes and found that arrays trapped the big particles with small migration angles, while small particles were free to move in the centrifugal force direction. Integrating deterministic lateral displacement (DLD) into the centrifugal platform not only improved the device portability by eliminating external pumping techniques and physical connections but also facilitated the preparation step for cell labeling and/or analysis using the platform.

### 3.8. Separation Based on Immunoaffinity Processes in Centrifugal Microfluidic Platforms

Separation based on or for immunoaffinity processes is another passive particle/cell isolation technique in the centrifugal microfluidic platform. Immunoaffinity is the utilization of surfaces or pre-activated particles with specific antigens/antibodies to isolate and sort specific target cells from heterogeneous background mixtures. Immunoaffinity is mostly integrated with other sorting mechanisms, including the density gradient medium (DGM) [197], capture-array [207], and waved microchannel for inertial sorting [209]. Figure 8e shows a fully automated centrifugal microfluidic system consisting of a blood chamber, a DGM chamber, and a collection chamber for the separation of CTCs from blood samples [197]. A triangular obstacle structure (TOS) was considered inside the blood chamber to avoid the backward movement of blood cells during the plasma evacuation process. Their design could deal with a relatively high volume of fresh blood (up to 5 mL) to separate CTCs without any preprocessing or using an external instrument. They showed their device’s ability by injecting 5 mL of a blood sample mixed with 100 µL of superparamagnetic activated microbeads with a diameter of 4.5 μm (used for CTC capturing) and then rotating the platform for a few minutes to separate plasma from the other blood components. The separated plasma was then transferred to the waste chamber, while the activated microbead-bound CTCs remained after a simple shaking process. Subsequently, the mixture was released to reach the DGM layer, where the CTC–bead complex descended into the collection chamber. They reported a high recovery rate of greater than 95%.

Previously, a centrifugal microfluidic platform was proposed to efficiently capture and analyze particles with bead-based assays (Figure 8f) [207]. This stopped-flow, sedimentation-based platform operated by injecting microparticles into the assigned chamber filled with stagnant fluid. While spinning the platform, the resultant centrifugal force pushed the randomly dispersed particles through the array of V-shaped traps. The authors performed a single-step antibody assay to demonstrate their device’s ability. Recently, Aguirre et al. used a zig-zag spiral geometry and integrated two operation units (a micromixer, to create breast cancer cells and bead complex MCF7-PS, and an inertial sorter, to separate the cancer–bead complex from the background mixture) onto the centrifugal platform [209] (Figure 8g). They designed the micromixer using the principle of secondary flow induced by the Dean drag force, whereas the cell sorter worked based on the lateral migration effect. The main objective of this design was to isolate MCF-7 from other blood cells. Before performing the separation process, the sample entered via the main inlet was combined with specific sticky micro-scaled particles over the mixing unit, which was intended to enhance the target cell volume and finally increase the separation efficiency. The process started by injecting the biological sample of MCF-7+DMEM culture media and the anti-EpCAM functionalized beads into inlets #1 and #2. Then, the platform was rotated at a specific angular velocity (3.75 Hz) to push the sample and microbeads into the micromixer. They reported a 97.1% mixing rate and a recovery rate of 98.7%, which made their device unique. Recently, a fully automated, WBC-negative depletion-based continuous centrifugal microfluidics-circulating tumor cell disk (CCM-CTCD) device was developed to capture heterogeneous CTCs [210].

### 3.9. Active Particle Separation Techniques in Centrifugal Microfluidics

**Figure 9:**
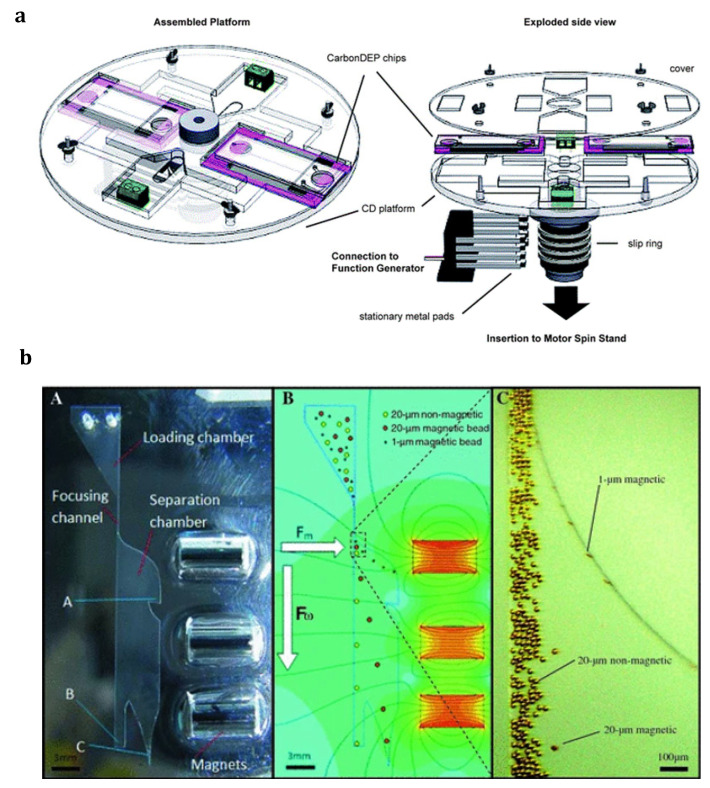

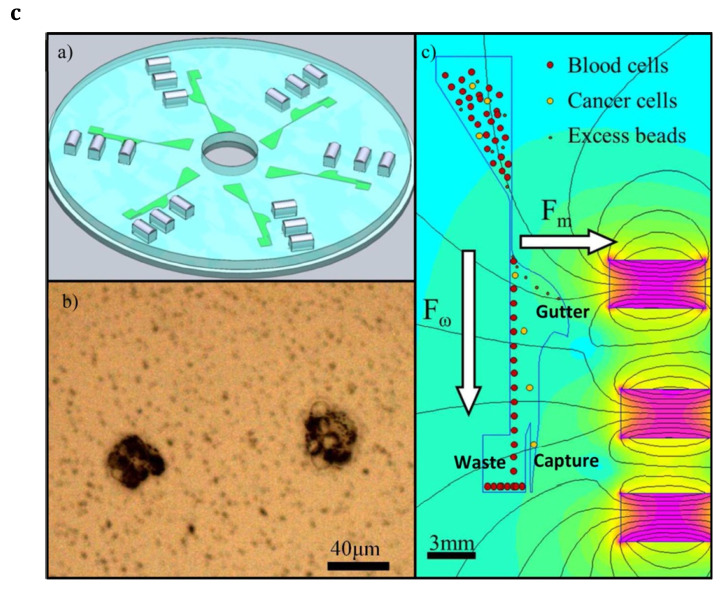
A centrifugal microfluidic system integrated with active separation techniques: (**a**) Integrated DEP on the centrifugal platform for selective particle/cell separation [211] (Reprinted/adapted with permission from Ref. [211]. 2010, Royal Society of Chemistry); (**b**) Particle/cell sorting consists of different chambers under the effect of centrifugal and magnetic forces [A shows a single centrifugo-magnetophoretic separation system with loading and separation chambers and a focusing channel on a rotating disc, B shows the trajectories and destinations of three different particles under the effect of separation forces (i.e., centrifugal, Coriolis and magnetic forces), and C shows the separated beads in different routes] [212] (Reprinted/adapted with permission from Ref. [212]. 2012, Springer Nature); (**c**) Schematic showing the polymeric LOCD with microfluidic channels (colored in green) and magnets (specified with silver cylinders) [a displays an overview of a polymeric LOCD with microfluidic channels and magnets, b shows two clusters of cancer cells capped with paramagnetic beads, and c shows the trajectories and destinations of blood cells, magnetically tagged cancer cells and excess magnetic beads under the effect of separation forces (i.e., centrifugal and magnetic forces) on a CCW rotating disk] [213] (Reprinted/adapted with permission from Ref. [213]. 2014, John Wiley and Sons).

After the main passive particle/cell separation methods on centrifugal microfluidic platforms emerged, active centrifugal microfluidics was also introduced. Unlike the passive separation techniques that work based on microfluidic geometry and physical properties of the particles, active separation techniques (i.e., dielectrophoresis, acoustophoresis, and magnetophoresis) use external forces to help the separation process [173,198]. Martinez-Duarte et al. implemented the dielectrophoresis approach on the centrifugal microfluidic platform to actively filter and capture the target cells [211] (Figure 9a). They fabricated and integrated 3D carbon electrodes with a voltage supply, resulting in a high filtration and separation efficiency and a low fabrication cost. Later, Kirby et al. and Siegrist et al. imposed a magnetic field on a centrifugal platform for cell manipulation using the stopped-flow sedimentation method [212,213] (Figure 9b). Their devices contained a loading chamber, a focusing channel, a fork-shaped separation chamber dividing various cell types into different destination sub-chambers under the effect of Stokes’ drag, centrifugal, and magnetic forces, and destination chambers A, B, and C. In independent research, Kirby et al. used functionalized magnetic beads with anti-EpCAM antibodies to separate rare MCF-7 cancer cells from a whole blood sample (Figure 9c). As shown in Figure 9c, the three governing forces (radial centrifugal force, lateral magnetic force, and hydrodynamic Stoke’s drag force) determine the path of the particles through the system. As expected, non-magnetic background blood cells were only affected by the centrifugal force and Stoke’s drag force (which is in the counter direction of centrifugal force). They finally sedimented on a straight path toward the CD’s peripheral edge. In the meantime, the magnetically tagged cancer cells not only experienced these two mentioned forces but also experienced lateral magnetic force. Therefore, they diverted from the mainstream toward the capture chamber. Kirby et al. achieved a recovery rate of 90–96% using their centrifugo-magnetophoresis device [213].

### 3.10. Limitations and Challenges of Centrifugal Microfluidic Systems for Cell Separation Applications

Despite all the mentioned advantages of centrifugal microfluidic systems, they have some disadvantages. The most undesirable limitation, originating from the low volume and small sample quantity, is that this method is unsuitable for isolating scarce cells [91]. For instance, this system has difficulty separating CTCs from a patient’s blood sample in one step. Therefore, it must always be used after a cell enrichment step or after an initial separator, which has concentrated the target cells. Although an initial separation outside the device might concentrate the target cells and eliminate this problem, the condensed sample might be contaminated during the time-consuming and sensitive transfer process from the first separator to the centrifugal microfluidic device. A solution to this issue could be integrating the first and second separation steps into a single disk, which would allow the sample to be concentrated in the first step and transferred to the second step without any contamination. However, placing two separation units on a single platform requires an innovative design that takes into account the location of units in radial distances, the execution possibility using available microfabrication techniques, and the interaction between existing forces. The existing forces must interact in such a way that in addition to flowing the fluid inside the microchannel, they also perform the process of particle separation. For this purpose, after the introduction of two novel types of passive microfluidics, it is better to become acquainted with the forces present in a microfluidic system.

## 4. Forces on Particles in a Microfluidic Device

In order to numerically model and experimentally investigate microfluidic devices in the field of cell separation, it is important to become familiar with the forces applied to cells in a microfluidic device. The movement trajectory of particles inside a microchannel can be obtained using the Lagrangian approach. From the Lagrangian point of view, Newton’s second law for particles is written in Equation (1):

(1) $$m_{p}\frac{du_{p}}{dt}=\sum \vec{F_{p}}=F_{D}+F_{L}+F_{P}+F_{G}+F_{Bu}+F_{Br}+F_{Am}+F_{Ba}\;+F_{Ext}+F_{Cen}+F_{Eu}+F_{Co}\;\;\;$$

where $\left(u_{p}\right)$ is the particle velocity vector, $\left(m_{p}\right)$ represents particle mass, and ($\sum \vec{F_{p}})$ is the resultant vector of the forces acting on the particle. $\left(F_{D}\right)$, $\left(F_{L}\right)$, $\left(F_{P}\right)$, $\left(F_{G}\right)$, $\left(F_{Bu}\right)$, $\left(F_{Br}\right)$, $\left(F_{Am}\right)$, $\left(F_{Ba}\right)$, $\left(F_{Ext}\right)$, $\left(F_{Cen}\right)$, $\left(F_{Eu}\right)$, and $\left(F_{Co}\right)$ are the drag, lift, pressure gradient, gravity, buoyant, Brownian, added mass, Basset, external field (if available), centrifugal, Euler, and Coriolis forces, respectively, each of which is explained below. Note that external fields include electric, acoustic, and magnetic fields. Furthermore, the centrifugal, Euler, and Coriolis forces are only included when the microfluidic device rotates at a specific angular velocity, like centrifugal microfluidics.

It should be noted that the relationships presented for the forces resulting from fluid flow to particles are a function of the particle dimensionless Reynolds number (or the so-called relative Reynolds number). Indeed, the two dimensionless Reynolds numbers can be defined using a fluid flow in a closed channel [214]. First, the Reynolds number $\left(Re=\frac{\rho UD_{h}}{\mu }\right)$ determines the flow type. Second, the particle Reynolds number includes the parameters describing the particle conditions in the fluid in which it flows. This number is defined as follows:

(2) $$Re_{p}=\frac{\rho d_{p}\left|u-u_{p}\right|}{\mu }=\frac{d_{p}{}^{2}}{D_{h}{}^{2}}Re=\frac{\rho Ud_{p}{}^{2}}{\mu D_{h}}$$

In Equation (2), ρ, $d_{p}$, μ, u, $u_{p}$, $D_{h}$, and Re are the fluid density, particle diameter, fluid viscosity, fluid velocity, particle velocity, channel hydraulic diameter, and flow Reynolds number, respectively. The particle Reynolds number can be defined using the average flow velocity in the channel ($Re_{p}=\frac{2}{3}\;Re)$ [214]. According to this calculated number, the mentioned forces are reviewed and applied in two different cases:

- Case #1: If $Re_{p}<1$, the fluid flow around the particle acts in a similar way to the creeping flow.
- Case #2: If $1<Re_{p}<100$, fluid inertia significantly affects the forces acting on a particle.

Although some attempts have been made to cover a more extensive range of relative Reynolds numbers, it is not necessary to mention them here due to not reaching these ranges in most case studies. After calculating the average and the maximum value or the particle Reynolds number within the channel route, the appropriate relations provided for this range of particle Reynolds numbers should be used to apply the drag and lift forces.

The following describes all the forces acting on the particles in a microfluidic system.

### 4.1. Drag Force

**Figure 10:**
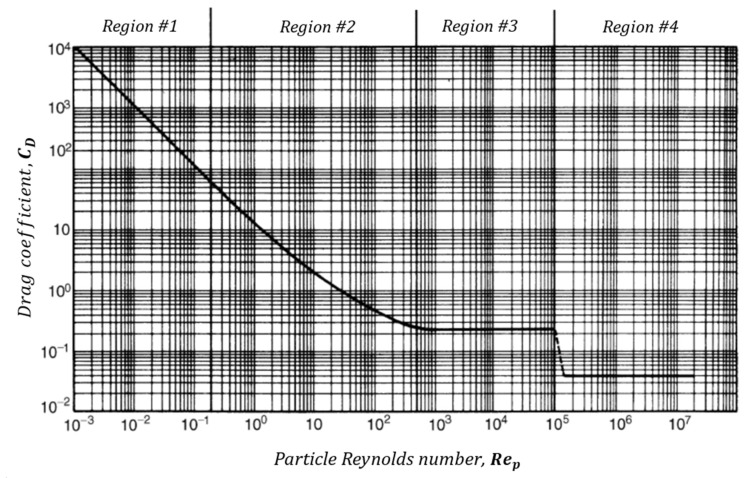
Drag coefficient according to the particle Reynolds number for a spherical particle [215] (Reprinted/adapted with permission from Ref. [215]. 2002, Elsevier).

Like any moving object in a fluid flow, the fluid applies a drag force to the particles against their movement direction. Indeed, in terms of fluid dynamics, the drag force acts opposite to the relative motion of particles moving with respect to the surrounding fluid (when the particle moves along the fluid or when the fluid moves relatively with respect to the particle). The amount of drag force depends on the particle Reynolds number. The relationship between the drag coefficient $C_{D}$ and the particle Reynolds number $Re_{p}$ can be easily obtained using a logarithmic diagram (Figure 10) [215]. This diagram can be divided into four regions based on $Re_{p}$ values.

For Region #1 $\left(10^{-4}<Re_{p}<0.2\right)$: In this area, the relationship between $C_{D}$ and $Re_{p}$ is a straight line with a slope of −1. As such, the drag force is calculated using Equation (3):

(3) $$F_{D}=\frac{1}{\tau_{p}}m_{p}\left(u-u_{p}\right)$$

In Equation (3), $\tau_{p}$ is defined as follows:

(4) $$\tau_{p}=\frac{\rho_{p}d_{p}^{2}}{18\mu }$$

where $F_{D}$ is the drag force, $m_{p}$ is the particle mass, $u_{p}$ is the particle velocity vector, u is the velocity vector of the fluid, and $\tau_{p}$ represents the particle relaxation time. One-third of this force is the contribution of drag force caused by pressure (pressure drag force), whereas two-thirds is the contribution caused by surface friction (friction drag force). It can be concluded from Equation (2) that the drag force in creeping flow (or Stoke’s flow) is only proportional to the velocity. Therefore, it is also known as Stoke’s drag force, which is only valid at very low particle Reynolds numbers, and its divergence increases continuously as the particle Reynolds number increases [216]. According to Stoke’s law, $C_{D}$ for a spherical particle is defined in Equation (5), which is only applicable at low particle Reynolds numbers.

(5) $$C_{D}=24\frac{\mu }{\rho ud_{p}}=\frac{24}{Re_{p}}$$

where $Re_{p}$ is the particle Reynolds number. As a result, the total force on the particle can be calculated as $F_{D}=3\pi \mu d_{p}u$.

For Region #2 $\left(0.2<Re_{p}<500\sim 1000\right)$: In this range, the slope of the curve gradually increases from −1 to 0 along with the increase in $Re_{p}$. If the flow regime around the particle is laminar (not creeping), the drag force should be modified due to the effect of fluid inertia. However, after calculating the particle Reynolds number, one can realize whether this modification is necessary or not. In this specific case, Schiller and Naumann’s modification is proposed. The drag force coefficient under Schiller and Naumann’s suggestion is defined in Equation (6) [217]:

(6) $$\tau_{p}=\frac{4\rho_{p}d_{p}^{2}}{3\mu C_{D}Re_{p}}$$

where $Re_{p}$ is the particle Reynolds number and $C_{D}$ is defined in Equation (7):

(7) $$C_{D}=\frac{24}{Re_{p}}\left(1+0.15Re_{p}{}^{0.678}\right)$$

As a result, the total force on the particle can be calculated as $F_{D}=3\pi \mu d_{p}u\left(1+0.15Re_{p}{}^{0.678}\right)$. The other two regions are in the range of turbulent flow and are outside the scope of this discussion.

### 4.2. Lift Force

Under suitable conditions, when the particles are randomly distributed in a laminar flow at a straight microchannel entrance, they will relocate and create a narrow ring-shaped structure at a distance of approximately 0.6 times the tube radius from the axis [92]. This phenomenon shows that apart from the drag force in the flow direction, additional lateral forces are applied to the particles, making them resettle and concentrate at specific lateral positions. Since flow inertia is the main source for creating the lateral forces inside the channel, they are usually known as inertial lift forces [94]. It can be shown that the walls play a significant role in this phenomenon. Without channel walls, the flow will be uniform, and no velocity gradient will be developed to induce rotational movement on a sphere; thus, no lateral lift force emerges.

**Figure 11:**
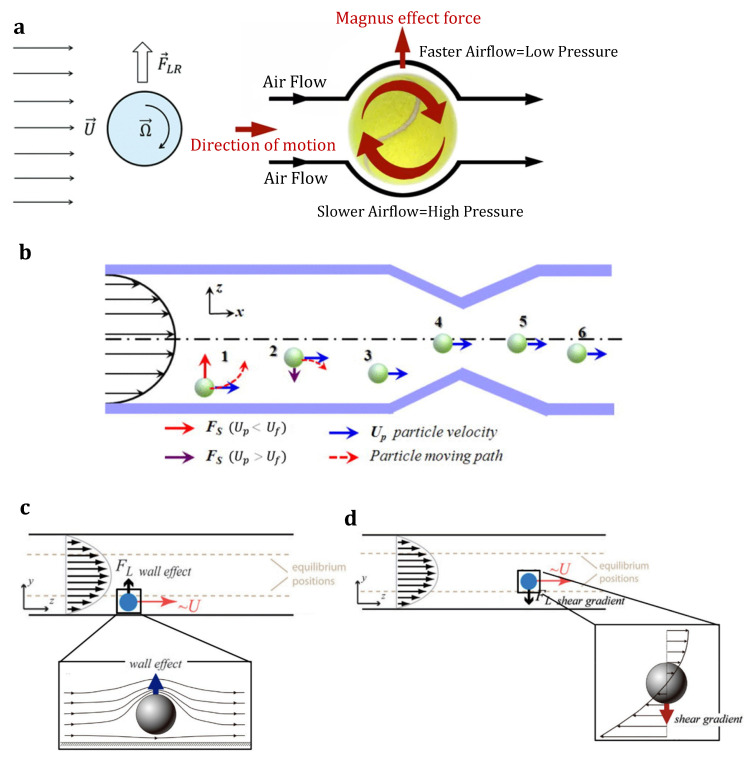
The four components of lift force: (**a**) The Magnus force (due to the interaction between slip and particle rotation at low Reynolds numbers) [96] (Reprinted/adapted with permission from Ref. [96]. 2016, Royal Society of Chemistry); (**b**) The Saffman force (caused by the interaction between slip velocity and shear) [223] (Reprinted/adapted with permission from Ref. [223]. 2012, AIP Publishing); (**c**) The wall lift force (arising from wall repulsion) [224]; (**d**) The shear gradient lift force [224].

Due to fluid inertia, the particles in a fluid flow are affected by force (perpendicular to the flow direction) consisting of four components:

I. The lift force originating from a rotating rigid cylinder or sphere/particle with a constant angular velocity (Ω) in an in viscous flow with a uniform velocity (u), known as the Magnus lift force [218] or rotation-induced lift force. This force is always directed toward the center of a channel [219]. Assuming a no-slip velocity condition on the sphere surface, the fluid velocity at the bottom part of the rotating particle with a constant angular velocity is lower than the velocity at the upper part. As a result, in terms of the Bernoulli principle, the pressure at the bottom of the sphere will be higher than at the upper part, which, in turn, leads to developing a lateral lift force in the direction of lifting the sphere due to the transverse pressure difference (Figure 11a). Indeed, the Magnus force can be viewed as a consequence of the pressure difference induced by the streamline asymmetry (or velocity field disturbance) caused by the rotation of a spherical object [220]. As shown on the right side of Figure 11a, in a real case study, the airflow above the ball moves quicker than the airflow below according to the ball’s spinning direction. Therefore, the pressure difference between the upper and lower part of the ball creates a lift force.

Equation (8) is used to calculate the magnitude of the Magnus lift force [96]:

(8) $$F_{Magnus\;}=\frac{1}{8}\pi d_{p}{}^{3}\rho \left(u-u_{p}\right)\times \Omega$$

where $\Omega =\Omega_{p}-0.5\nabla \times u$ represents the relative rotation between the fluid and the rotational sphere with an angular velocity ($\Omega_{p}$) in a rotational flow field, $d_{p}$ is the particle diameter, and ρ, u, $u_{p}$, and $\Omega_{p}$ are the fluid density, fluid velocity vector, particle linear velocity vector, and particle angular velocity vector, respectively. In the above equation, the particle is not stationary but is simultaneously moving through the fluid with a velocity of $u_{p}$. The direction of this force is perpendicular to the plane defined using the relative velocity vectors and the rotation axis [96]. It should be mentioned that the Magnus force is negligible compared to the other three components of the lift force because it has a very small value in low-velocity flows [221].

II. The presence of walls generates a fluid velocity gradient (shear rate) and shear-induced particle rotation, which makes the particle lag behind the fluid. This slip–shear motion causes a lateral force known as the Saffman lift force on the particles. The slip–shear-induced lift force, developed from the velocity gradients in the opposite direction, only applies to a sphere with a constant shear rate and a zero-shear gradient (i.e., an unbounded simple shear flow). A particle in a parabolic velocity field experiences a larger relative velocity on the side of the particle away from the parabola’s maximum point. Such a difference in the velocity profile creates a pressure difference that develops a force that always directs toward the side with a higher magnitude of relative velocity [219]. It should be noted that this force is the only shear rate effect that is independent of the particle rotation. In a real situation, the spheres move freely, and the shear induces a relative rotation. As a result, a Magnus force will appear on the sphere. The effects of shear and rotation cannot be considered independent in most practical flows [220]. If the angular velocity is not significantly greater than the shear rate, the Saffman force will be much greater than the Magnus force for a freely rotating particle [222]. Figure 11b shows that if the particles are leading the flow, the Saffman force points to the channel walls in a simple shear flow; if the particles are lagging the flow, the Saffman force directs to the channel centerline [96,223].

Equation (9) is used to calculate the magnitude of the Saffman force, which is the lateral lift force on a sphere in a simple unbounded shear flow:

(9) $$F_{saffman}=-81.2r_{p}{}^{2}L_{v}\sqrt{\mu \rho \frac{\left|u-u_{p}\right|}{\left|L_{v}\right|}}$$

where $F_{s}$, $r_{p}$, μ, ρ, u, and $u_{p}$ are the Saffman lift force, particle radius, fluid viscosity, fluid density, fluid velocity vector, and particle velocity vector, respectively. The value of $L_{v}$ is also calculated as $L_{v}=\left(u-u_{p}\right)\times \left[\nabla \times \left(u-u_{p}\right)\right]$.

III. Another effect of the existence of walls is that they disturb the flow field around the particle, especially when the particle is moving near the walls. When a particle moves near a wall, the constricted flow on the side of the particle close to the wall has more pressure than the opposite side. Therefore, this pressure difference causes a wall-induced lift force that pushes the particles away from the wall (Figure 11c) [219].

The wall-induced lift force is computed using Equation (10):

(10) $$F_{wall}=\rho \frac{r_{p}{}^{4}}{D^{2}}\beta \left(\beta G_{1}\left(s\right)+\gamma G_{2}\left(s\right)\right)n$$

where β and γ are:

(11) $$\beta =\left|D\left(n.\nabla \right)u_{II}\right|$$

(12) $$\gamma =\left|\frac{D^{2}}{2}{\left(n.\nabla \right)}^{2\;}u_{II}\right|$$

In these equations, $u_{II}=\left(I-n\times n\right)u$, s is the dimensionless distance between the particle and the first channel wall, n is the normal unit vector to this wall, D is the distance between the two walls, and I is the identity matrix. Each $G_{1}$ and $G_{2}$ function is a specific function defined based on the distance between the particle and the wall.

IV. The shear gradient-induced lift force on a particle appears from the curvature of the parabolic velocity profile in a Poiseuille flow with a non-zero shear gradient. Due to the parabolic nature of the velocity profile, the relative velocity magnitude of the fluid to the particle is much higher on the left side than on the right side of the particle (Figure 11d). This dissymmetry in relative velocity causes a pressure difference on two sides of the particle and develops a shear gradient lift force in the opposite direction of the wall-induced force. The shear gradient lift force leads particles to migrate toward the walls until reaching a balance position by repelling the wall-induced lift force [222].

It should be noted that the recently mentioned relationships for calculating the magnitude of lift forces are only acceptable and used when the particle Reynolds number is less than one ($Re_{p}<1$) (i.e., Case #1). If the particle Reynolds number increases ($Re_{p}>1$) (i.e., Case #2), modified relations should be used to calculate the total lift force.

**Figure 12:**
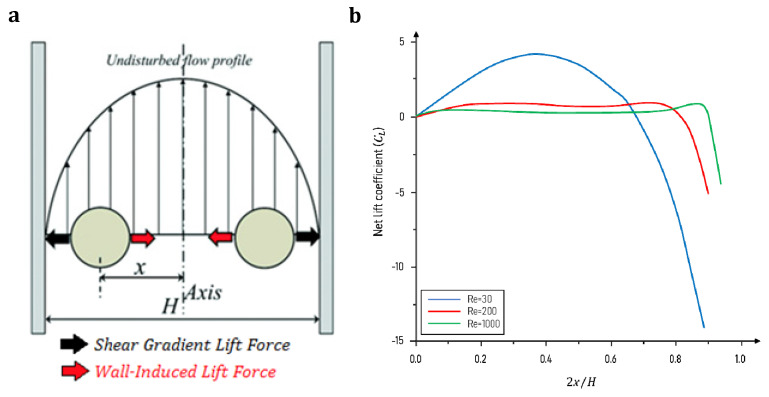
The position of a particle under the effect of the net lift force: (**a**) The balance between the recognized lift forces creates the inertial equilibrium position for the particle in the Poiseuille flow [96] (Reprinted/adapted with permission from Ref. [96]. 2016, Royal Society of Chemistry); (**b**) The net lift coefficient ($C_{L}$) as a function of the Reynolds number and the particle lateral position [226].

In addition, it was mentioned that for a neutrally buoyant rigid sphere flowing in a straight wall-bounded Poiseuille flow, in addition to a viscous drag force along the axis, four lateral forces are acting on the particle. Among them, the Saffman and Magnus forces are often minimal and negligible; therefore, the shear gradient lift force (directing particles toward the channel walls) and wall-induced lift force (repelling particles toward the channel centerline) are typically identified as the dominant effects in lateral particle migration [225] (Figure 12a). As a result of balancing the shear gradient lift force and wall-induced lift force, several halfway equilibrium positions are formed. Based on the current conditions, the relation (13) presented by Asmolov is appropriate for a small rigid sphere ($d_{p}/H<1$) in a Poiseuille flow [214]:

(13) $$F_{L\;}=\rho G^{3}C_{L}d_{p}{}^{4}$$

His findings determined that the $C_{L}$ coefficient value was a function of the particle’s dimensionless lateral position with respect to the microchannel center and the Reynolds number (Figure 12b) [226].

### 4.3. Pressure Gradient Force

Since the particles are immersed in the fluid flow, the pressure gradient force in the fluid is applied to them. This force is expressed in Equation (14):

(14) $${\vec{F}}_{P\;}=m_{f}\left(\frac{D\vec{u}}{Dt}-v\nabla^{2}\vec{u}\right)$$

In this equation, ${\vec{F}}_{P\;},$ $m_{f}$, and v are the force of the pressure gradient on the particle, the mass of the fluid displaced by the particle (equal to the fluid density multiplied by the particle volume), and the fluid kinematic viscosity, respectively.

### 4.4. Gravity Force

Gravity force is exerted on an object due to the field of gravitational attraction of the Earth. The amount of this vector force equals the object’s mass times the gravitational acceleration value (g) at that location. Its direction also aligns with the direction of gravitational acceleration (W=mg). The gravity force for a spherical particle is expressed in Equation (15):

(15) $$F_{G\;}=\frac{4}{3}\pi R^{3}\rho_{p}g$$

where $\rho_{p}$ is defined as particle density, and g is gravitational acceleration.

### 4.5. Buoyant Force

The buoyant force is the name of the upward force exerted on objects, whether it floats or sinks. Indeed, the buoyant force is applied to an object in the opposite direction of its acceleration (the situation of any object immersed in a fluid). Therefore, the buoyant force is related to pressure differences between a submerged object’s bottom and top. In a static fluid, pressure (P=ρgh) increases as the depth increases due to a greater fluid weight on the object. The downward force exerted by the pressure on the top of the object will be less than the upward force exerted by the pressure on the bottom of the object (because the bottom of an object is always deeper in a fluid than its top). This pressure difference creates a force that moves the object against its weight force direction. The value of this force is equal to the pressure difference between the bottom and top surfaces of the object. Based on Archimedes’ principle, the buoyant force on an object is equal to the weight of the fluid displaced by the object (or equal to the mass of the displaced fluid times the acceleration magnitude due to gravity). The buoyant force for a sphere submerged in a fluid is expressed in Equation (16):

(16) $$F_{Bu\;}=\rho_{f}V_{f}g=m_{f}g=W_{f}=\frac{4}{3}\pi R^{3}\rho g$$

where $V_{f}$, $\rho_{f}$, $m_{f}$, and $W_{f}$ are the volume, density, mass, and weight of the displaced fluid, respectively, and R is the sphere radius.

The result of the two forces of buoyancy and gravity that are applied to all particles is represented in Equation (17):

(17) $$F_{Bu,\;G\;}=\left(\rho_{f}-\rho_{p}\right)V_{p}g$$

In Equation (17), $\rho_{f}$, $\rho_{p}$, and $V_{p}$ are the fluid density, particle density, and particle volume, respectively.

### 4.6. Brownian Force

In physics, the random motion of particles in a fluid caused by their collisions with surrounding atoms and molecules is called Brownian motion. Brownian forces affect the motion of particles whose dimensions are smaller than the micrometer scale. Statistical calculations and probability functions are used to compute these forces. The spectral intensity for this process is defined in Equation (18) [227]:

(18) $$S_{ij}^{n}=S_{0}\delta_{ij}$$

where $S_{0}$ is defined in Equation (19):

(19) $$S_{0}=\frac{216vkT}{\pi^{2}\rho d_{p}{}^{5}S^{2}C_{c}}$$

In Equation (19), T is the fluid temperature, $d_{p}$ is the particle diameter, k is the Boltzmann’s constant, S is the ratio of the particle density to the fluid density, and $C_{c}$ is the Cunningham correction factor, which is defined in Equation (20) [228]:

(20) $$C_{c}=1+\frac{2\lambda }{d}\left(1.257+0.4e^{\left(-1.1d_{p}/2\lambda \right)}\right)$$

In Equation (20), λ is equal to the mean free path. Thus, the Brownian force is obtained using the following equation:

(21) $$F_{Br}=G\sqrt{\frac{\pi S_{ij}}{∆t}}$$

where ∆t is the time step, and the value of *G* is determined using statistical calculations. Since the behavior of particles with a diameter larger than 1 µm is studied in the rest of this section, the effects of Brownian force can be disregarded.

### 4.7. Added Mass Force

Another force that is applied to particles by the fluid is the added mass force. During an accelerated motion, the mass of the accelerating fluid is also important, along with the object’s mass. The fluid applies a force (called the added or virtual mass force) on the afloat particle due to the accelerated motion of the particle, which, in turn, requires a volume displacement of the fluid. In fluid mechanics, added mass or virtual mass is a kind of resistance that results from inertia that is added to the system due to displacing some volume of the particle’s surrounding fluid during its accelerating or decelerating movement. Indeed, added mass is defined because the object and surrounding fluid cannot simultaneously occupy the same physical space. Therefore, the movement of the immersed object increases the fluid kinetic energy. Although “all” the fluid will actually be accelerated to different degrees, this phenomenon can be simply modeled as some volume of fluid moving with the object. The magnitude of this force for a spherical object immersed in a non-viscous and incompressible fluid can be calculated using Equation (22) [229]:

(22) $$F_{Am}=\frac{\rho_{f}V_{p}}{2}\left(\frac{Du}{Dt}-\frac{Du_{p}}{Dt}\right)$$

where $\rho_{f}$ is the density of the fluid surrounding the particle and $V_{p}$ is the particle volume, which is equal to the displaced fluid volume owing to the accelerated motion of the particle in the fluid.

In many physical problems, added mass is calculated by considering the effective mass as the sum of the actual and added masses. Indeed, the sum of these two masses is usually known as the virtual mass. Based on the virtual mass, Newton’s second law can be written in the following simple form (Equation (23)):

(23) $$F=\left(m_{p}+m_{am}\right)a$$

where a is the particle’s acceleration vector and $m_{p}$ and $m_{am}$ represent the actual mass of the particle and the added mass, respectively. In this case, it can be shown that for a sphere with a radius R, the added mass amount equals $2/3\rho \pi R^{3}$. However, the added mass is generally written as a tensor whose components depend on the particle’s movement direction.

The added mass force has an effect on all particles that accelerate in a fluid. However, due to the dependency of the added mass on the fluid density, this effect is negligible for dense particles in a low-density fluid. This force becomes important when the fluid density is greater than or close to the particle density. Based on the characteristics of particles and fluid considered in this review, particles and fluid have almost equivalent densities. This means that the effects of this force must be regarded while studying the behavior of cells.

### 4.8. Basset Force

As the particle acceleration affects its surrounding fluid and causes the added mass force, the viscous effects of the surrounding fluid also affect the accelerating particle under the Basset force. This force is induced due to the delay in the development of the boundary layer when the velocity changes with time. For a spherical particle immersed in a fluid, the Basset force can be calculated using Equation (24) [230]:

(24) $$F_{Ba,\;i}=\frac{3}{2}d_{p}{}^{2}\sqrt{\pi \rho \mu }\int_{0}^{t}\frac{\frac{d}{dt^{\prime }}\left(u_{i}-u_{p,i}\right)}{\sqrt{t-t^{\prime }}}dt^{\prime }$$

As can be seen from the definition, the effects of this force will be substantial when the changes with time are significant and varied. For example, the Basset force is considered in calculations for a 10 µm diameter particle in a flow oscillating with a frequency of 700 Hz [230]. On the other hand, this effect can be neglected in the current simulation due to the absence of large oscillations in the fluid flow inside the microchannel.

### 4.9. Forces on Particles in a Rotational Platform

Since a system with microchannels mounted on a rotating platform is studied in this paper, the volumetric forces induced by the rotation of the CD, in addition to the previously mentioned classic forces, are applied to the particles in the fluid. As the fluid itself experiences centrifugal, Coriolis, and Euler forces in centrifugal microfluidic systems, these forces are also applied to the particles immersed in that fluid. The centrifugal, Euler, and Coriolis forces are defined in Equations (25)–(27):

(25) $$F_{Cen,p}=\left(\rho_{f}-\rho_{p}\right)V_{p}\vec{w}\times \left(\vec{w}\times {\vec{r}}_{p}\right)$$

(26) $$F_{Eu,p}=m_{p}{\vec{r}}_{p}\times \frac{dw}{dt}$$

(27) $$F_{Co,p}=2m_{p}\vec{w}\times {\vec{u}}_{p}$$

In these equations, $m_{p}$ represents the mass of each particle. At a constant angular velocity and a specific distance of r, the amount and intensity of the centrifugal force are much more effective than the other two forces, which, in turn, causes it to be the primary driving factor for the fluid flow inside the microchannel. Therefore, channels and microfluidic systems are often located radially to supply their propulsion force. The Coriolis force on the fluid flow is applied perpendicular to the rotation direction in the opposite direction of system rotation, whereas the Euler force is applied perpendicular to the rotation direction but in the direction of the system rotation. These two forces are most influential in cases of retarded and accelerated speed (change in angular velocity). It should be noted that all these forces can be controlled by changing the angular velocity. For example, changing the channel width and using these forces can control the angular velocity in such a way as to stop the fluid flow (which is the fundamental concept of a passive microfluidic valve in rotational platforms).

## 5. Conclusions

Separating cells based on their biochemical or physical properties can be performed with advantages and disadvantages. Among the size and density-based separation techniques, inertial microfluidics plays a significant role in the separation field. This is because of their (1) relatively large dimensions, (2) simple design and fabrication, (3) high throughput, (4) simple experimental setup, (5) high recovery rate, and (6) high operational flow rate. Generally, inertial microfluidics is more suitable than centrifugal microfluidics for processing high-volume samples, such as for separating CTCs, because loading the samples with syringe pumps can provide a broader range of required sample volumes. Numerous studies have shown that spiral microfluidic designs can be easily adapted to different applications by modifying the spiral cross-sectional area, dimension, length, and flow rate. Meanwhile, multi-orifice designs with various structures, including a single chamber (single vortex), multi-chambers on a single side of the channel, and multi-chambers on both sides of the channel, have been developed to suit specific applications. As an all-rounder, the inertial contraction–expansion structure has been used for particle manipulation, especially since introducing the secondary flow further improves the manipulation’s efficiency. The Dean flow in the contraction–expansion microchannel can drastically reduce the channel length and processing time. As a result, inertial microfluidic devices with various structures would be highly recommended for the initial filtration of large volumes of blood samples in a compact format to improve separation efficiency.

On the other hand, it suffers from some weaknesses: (1) maintaining the flow rate in a specific range to reach a suitable separation, (2) pump requirements to drive the fluid, (3) using a syringe pump decreases the platform’s flexibility for combining preprocessing steps in more involved procedures, (4) low control over cell movement, (5) the possibility of clogging, and (6) no ability to control the operation precisely. Therefore, despite the fact that such rapid separation systems are valuable for increasing purity and yield, due to the above-mentioned disadvantages, researchers have started thinking about using centrifugation approaches.

Centrifugation approaches use the physical centrifugation process on a rotational microfluidic platform, such as in LOCD, or strategies that use the liquid centrifugation effect resulting from the Dean effect for cell/particle separation, such as in spiral and multi-orifice microfluidics. Particle/cell separation strategies using centrifugal microfluidic devices can be chosen between passive and active separation technologies or even a combination of those. Most studies in this field indicated that the centrifugal microfluidic platform is most appropriate when a raw sample preparation step is required to be integrated on the same platform. The significant benefits of using the centrifugal microfluidic platforms to separate particle/cell are (1) a simple design and fabrication process, (2) ability to integrate multi-processing stages of mixing, valving, centrifugation, etc., (3) improved portability of the proposed methods, (4) less human interaction, and (5) contain a wide range of implementable unit/operations that can be used in various applications.

As both centrifugal and inertial microfluidic platforms have their benefits and drawbacks, the application requirement should be carefully considered before platform selection. For instance, when designing a point-of-care application for a low-resource environment requires preprocessing, centrifugal microfluidic devices are preferable. In contrast, if the intention is to miniaturize the commercially available appliances with reduced processing time and a relatively high sample volume, then an inertial microfluidic platform is the better choice. However, a combination of centrifugal and inertial microfluidic platforms can strengthen their advantages and cover each method’s disadvantages. It is obvious that such an idea needs adequate knowledge of different separation techniques, the microfabrication process, and their restrictions in both simulation and fabrication aspects. For example, the main restrictions to using centrifugal microfluidic platforms for cell separation are the limitation of processing a large volume of samples and reagents and the limited space for installing components and units, which requires a precise design and consideration. Although some authors claimed the ability to process around 5 mL sample volume using centrifugal microfluidic devices, processing such volumes would limit the number of processes that could be multiplexed on the same platform. The authors hope the information given in this review paper will help researchers understand the interaction between fluid and particles moving within the microchannel and have a better understanding of the mechanisms in a microfluidic device used in the simulation and experimental works. Table 1 shows as overall comparison of papers published in the last five years in the field of cancer cell separation with the help of inertial and centrifugal microfluidic devices.

## Figures and Tables

**Table 1:** Overall characteristics of inertial and centrifugal microfluidic cell separating devices developed in the last five years.

Separation Techniques	Method	Applied Forces	Shape of Channels	Type of Particles	Separation Efficiency (%)	Type of Microfabrication	Year	Ref.
Inertial microfluidics	Size-based inertial particle sorting	Inertial lift forces and Dean drag force	Wavy microchannels with a reverse curvature	Breast cancer cells (MCF-7) from diluted whole blood samples	89.72%	Soft lithography	2018	[89]
Inertial microfluidics	Inertial and deformability principles	Inertial lift and viscous drag forces; Dean forces and centrifugal forces	Triplet parallelizing spiral microchannels	Breast cancer cells (MCF-7) from diluted mimic patient blood	90%	Soft lithography	2018	[115]
Inertial and vortex microfluidics	The combined effects of inertial and vortex forces	Inertial lift forces and Dean vortices	Asymmetric contraction–expansion microchannel with sharp corners	Polystyrene particles	Not stated	Soft lithography	2018	[136]
Inertial microfluidics	Size-based inertial particle sorting	Inertial lift forces and Dean vortices	Rectangular microchannels with different contraction–expansion ratios	Polystyrene particles	Not stated	Numerical simulations	2018	[154]
Hybrid microfluidic method	Coupling inertial microfluidics with deterministic lateral displacement (DLD)	Inertial lift forces for size-based separation of particles; DLD-generated steric and hydrodynamic interaction forces for further separation	Spiral channel in the first stage, microchannels with a series of obstacles for DLD, and trapezoidal microchannels with alternating expansion–contraction regions for inertial microfluidics	Polystyrene beads and Breast cancer cells (MCF-7)	>99.9% for polystyrene beads; 91.34% for MCF-7 cells from leukocytes	Soft lithography	2019	[103]
Inertial microfluidics	Size-based inertial particle sorting	Inertial lift forces and Dean drag forces	A symmetrically curved channel	MDA-MB-231, Jurkat, K562, and HeLa	>90%	Soft lithography	2019	[107]
Inertial microfluidics	Size-based inertial particle sorting	Inertial lift forces and Dean drag forces	Contraction–expansion array channels	Polystyrene microspheres, plasma, RBCs, and NCI-H1299 cancer cells from blood	>95% for NCI-H1299 cells; 96.0% for Plasma and RBCs	UV laser system	2019	[139]
Inertial microfluidics	Size-based inertial particle sorting	Shear and lift forces	Spiral and contraction–expansion array	Leukocytes	Cell viability was investigated	Soft lithography	2019	[155]
Inertial microfluidics	Size-based inertial particle sorting	Inertial lift force, hydrodynamic drag force, and Dean drag force	Fishbone-shaped channel	Human brain malignant glioma cells (U87)	≥90%	Soft lithography	2020	[114]
Inertial microfluidics	Size-based inertial particle sorting	Inertial lift force, and Dean drag force	Spiral microchannel with right-angled triangular cross-section	Fluorescent microbeads and DU145 cells (human prostate cancer cell line)	Not stated	DLP/SLA 3D printer	2020	[117]
Inertial microfluidics	Self-amplified inertial focusing (SAIF)	Inertial lift force	A narrow zig-zag channel connected with two expansion regions	Lung cancer cells (A549), breast cancer cells (MCF-7), and cervical cancer cells (HeLa) from WBCs	65.6% for A549, 79.1% for MCF-7, and 85.4% for HeLa	Soft Lithography	2020	[120]
Inertial microfluidics	Size-based inertial particle sorting	Inertial lift force; Dean flow drag force	Channel with embedded microsquares along a series of repeating curved units	Two species of Candida (Cornus glabrata and Candida albicans) from Candida-spiked blood samples	>80%	Soft Lithography	2020	[157]
Inertial microfluidics	Size-based inertial particle sorting	Vortex-induced lift force; Dean drag force	Spiral channel with periodic expansion structures	MCF-7, Hela, and A549 cells	93.5% for MCF-7, 89.5% for Hela, and 88.6% for A549	Soft Lithography	2020	[158]
Inertial-ferrohydrodynamic microfluidics	Size-based inertial particle sorting and ferrohydrodynamic separation	Inertial lift force, and ferrohydrodynamic force	Narrow zig-zag microchannel connected to expansion sites	Two human breast cancer cell lines (MCF-7 and MDA-MB-231) and two human lung cancer cell lines (H1299 and H3122)	97.2 ± 4.0% for H1299, 91.8 ± 4.3% for MDA-MB-23, 95.4 ± 5.1% for MCF-7, and 94.6 ± 3.1% for H3122	Soft Lithography	2021	[121]
Integrated microfluidic method	Hydrodynamic inertial focusing and dielectrophoretic separation	Inertial lift force, Dean drag force, and dielectrophoretic force	Curved channel	RBCs and platelets (size between 2 and 4 µm) from CTCs and leukocytes (9–12.2 µm)	99.5%	Numerical simulations	2022	[147]
Hybrid microfluidic method	Dean flow fractionation (DFF) separation method	Inertial lift force and Dean drag force	Double-spiral channel	A549 cancer cells from RBCs and WBCs	87%	Soft lithography	2022	[119]
Integrated microfluidic method	Hydrodynamic inertial focusing and dielectrophoretic separation	Inertial lift force, DEP force, and alternating curvature-induced Dean force	Curved channel	MDA-231 CTCs from identical-sized WBCs and RBCs	86.7%	Soft lithography	2022	[122]
Inertial microfluidics	Centrifuge based on inertial microfluidics	Inertial lift force and centrifugal force	Four parallel inertial spiral channels and a two-stage serpentine channel connected in series	Breast cancer cells (MCF-7), lung cancer cells (A549) from a calcein-AM staining solution, and WBCs from lysed whole blood	>93%	Soft lithography	2022	[231]
Integrated microfluidic method	Hydrodynamic inertial focusing and acoustophoretic separation	Inertial lift force, DEP force, and acoustic radiation forces	Serpentine channel	Breast cancer cells (MCF-7) from neutrophil WBCs	99.3%	Numerical simulations	2023	[101]
Centrifugal microfluidics	Centrifugal nanoparticles separation and extraction (μCENSE) platform	Centrifugal forces	Serpentine channel with single inlet and two bifurcating outlets	Isolation of microvesicles	~90%	Soft lithography	2018	[232]
Centrifugal microfluidics	Inertial particle focusing on a centrifugal platform	Secondary flow drag force, inertial lift force, and centrifugal forces	Serpentine channel with three corner angels	Polystyrene particles	Not stated	Numerical simulations	2018	[233]
Integrated Centrifugal microfluidics	Inertial particle focusing on a centrifugal platform	Inertial force and centrifugal force	Contraction–expansion microchannel arrays	Polystyrene particles	~90%	Numerical simulations	2020	[234]
Centrifugal microfluidics	Inertial particle focusing on a centrifugal platform	Inertial force and centrifugal force	Straight channels and integrated trapezoidal microchambers	5 and 10 μm polystyrene fluorescent particles	~92% for 5 μm particles and ~98% for 10 μm particles	Femtosecond laser ablation	2020	[235]
Centrifugal microfluidics	Centrifugation; separator gel-based blood-plasma separation	Inertial force and centrifugal force	Rectangular channel	Blood cells and plasma	99.992%	Laser-cutting machine	2022	[236]
Centrifugal microfluidics	Centrifugal platform for the inertial separation	Inertial force and centrifugal force	Contraction–expansion array microchannels	Polystyrene particles	100%	Numerical simulations	2022	[237]
Centrifugal microfluidics	Density gradient centrifugation with WBC-negative depletion	Centrifugal force	An upper plate, which includes the wax valves, bonded to a lower plate	Lung cancer cells (PC-9, A549, H1975, and H1688), breast cancer cells (MDA-MB-231, SK-BR-3, and MCF-7), and bladder cancer cell (T24)	>90%	CNC milling machine	2022	[210]
Centrifugal microfluidics	Multiple single-cell retrieval strategy	Centrifugal force	Five-layer PMMA consisting of three spiral microchannels	Trophoblasts from maternal circulation	91.0%	CNC machining	2023	[238]

## Data Availability

Not applicable.

## Nomenclature

**Parameter**	**Name**
*AR*	Channel aspect ratio
$C_{c}$	Cunningham correction factor
$C_{D}$	Drag coefficient
$C_{L}$	Net lift coefficient
D	Distance between two channel walls
De	Dean number
$D_{h}$	Channel hydraulic diameter
d	Diameter
$d_{p}$	Particle diameter
$F_{Am}$	Virtual mass force
$F_{Ba}$	Basset force
$F_{Br}$	Brownian force
$F_{Bu}$	Buoyant force
$F_{Cen}$	Centrifugal force
$F_{Co}$	Coriolis force
$F_{D}$	Drag force
$F_{Eu}$	Euler force
$F_{Ext}$	External field force
$F_{G}$	Gravity force
$F_{L}$	Lift force
$f_{L}$	Dimensionless lift coefficient
$F_{LS}$	Shear gradient inertial lift force
$F_{LW}$	Wall-induced inertial lift force
$F_{P}$	Pressure gradient force
$F_{s}$	Saffman lift force
g	Volumetric force per unit mass; Gravitational acceleration
H	Channel height
k	Boltzmann’s constant
$m_{am}$	Added mass
$m_{f}$	Mass of displaced Fluid
$m_{p}$	Particle mass; Actual mass of particle
n	Normal unit vector
p	Pressure
R	Sphere radius
Re	Reynolds number
$Re_{p}$	Particle Reynolds number
r	Radial axis
$r_{c}$	Curvature radius of channel
$r_{p}$	Particle radius
S	The ratio of particle density to fluid density
T	Fluid temperature
t	Time
u	Fluid velocity
$u_{p}$	Particle velocity
$V_{f}$	Volume of displaced fluid
$V_{p}$	Particle volume
v	Fluid kinematic viscosity
W	Channel width
$W_{f}$	Weight of displaced fluid
λ	Mean free path
μ	Fluid dynamic viscosity
ρ	Fluid density
$\rho_{f}$	Density of displaced fluid
$\rho_{p}$	Particle density
ω	Angular velocity
$\Omega_{p}$	Particle angular velocity
$\tau_{p}$	Particle relaxation tim
